# The Wnt Pathway Inhibitor RXC004 Blocks Tumor Growth and Reverses Immune Evasion in Wnt Ligand–dependent Cancer Models

**DOI:** 10.1158/2767-9764.CRC-21-0095

**Published:** 2022-09-02

**Authors:** Caroline Phillips, Inder Bhamra, Catherine Eagle, Eimear Flanagan, Richard Armer, Clifford D. Jones, Matilda Bingham, Peter Calcraft, Alicia Edmenson Cook, Ben Thompson, Simon A. Woodcock

**Affiliations:** 1 Redx Oncology Ltd, Redx Pharma PLC; Cheshire, United Kingdom.; 2 Concept Life Sciences Ltd, Manchester, United Kingdom.; 3 Analytical Development, Flu-BPD, AstraZeneca PLC, Manchester, United Kingdom.; 4 Oncology Cell Therapy, GlaxoSmithKline PLC, London, United Kingdom.; 5 In Vitro, RxCelerate Ltd, Cambridge, United Kingdom.

## Abstract

Wnt signaling is implicated in the etiology of gastrointestinal tract cancers. Targeting Wnt signaling is challenging due to on-target toxicity concerns and lack of druggable pathway components. We describe the discovery and characterization of RXC004, a potent and selective inhibitor of the membrane-bound *o*-acyl transferase Porcupine, essential for Wnt ligand secretion. Absorption, distribution, metabolism, and excretion and safety pharmacology studies were conducted with RXC004 *in vitro*, and pharmacokinetic exposure assessed *in vivo*. RXC004 effects on proliferation and tumor metabolism were explored in genetically defined colorectal and pancreatic cancer models *in vitro* and *in vivo*. RXC004 effects on immune evasion were assessed in B16F10 immune “cold” and CT26 immune “hot” murine syngeneic models, and in human cell cocultures. RXC004 showed a promising pharmacokinetic profile, inhibited Wnt ligand palmitoylation, secretion, and pathway activation, and demonstrated potent antiproliferative effects in Wnt ligand–dependent (*RNF43*-mutant or *RSPO3*-fusion) colorectal and pancreatic cell lines. Reduced tumor growth and increased cancer cell differentiation were observed in SNU-1411 (*RSPO3*-fusion), AsPC1 and HPAF-II (both *RNF43*-mutant) xenograft models, with a therapeutic window versus Wnt homeostatic functions. Additional effects of RXC004 on tumor cell metabolism were confirmed *in vitro* and *in vivo* by glucose uptake and ^18^fluorodeoxyglucose-PET, respectively. RXC004 stimulated host tumor immunity; reducing resident myeloid-derived suppressor cells within B16F10 tumors and synergizing with anti-programmed cell death protein-1 (PD-1) to increase CD8^+^/regulatory T cell ratios within CT26 tumors. Moreover, RXC004 reversed the immunosuppressive effects of HPAF-II cells cocultured with human peripheral blood mononuclear cells, confirming the multiple anticancer mechanisms of this compound, which has progressed into phase II clinical trials.

**Significance:**

Wnt pathway dysregulation drives many gastrointestinal cancers; however, there are no approved therapies that target the pathway. RXC004 has demonstrated the potential to block both tumor growth and tumor immune evasion in a genetically defined, clinically actionable subpopulation of Wnt ligand–dependent gastrointestinal cancers. The clinical utility of RXC004, and other Porcupine inhibitors, in such Wnt ligand–dependent cancers is currently being assessed in patient trials.

## Introduction

Wnt pathway aberrations are common in gastrointestinal cancers (1–3). The canonical Wnt pathway is activated via canonical Wnt ligands (e.g., Wnt3a) binding to Frizzled receptors coupled to low-density lipoprotein receptor–related protein (LRP) 5 or 6, and leads to stabilization of β-catenin by disrupting the inhibitory complex of adenomatous polyposis coli (APC), Axin and glycogen synthase kinase-3β, with subsequent activation of transcription factor and/or lymphoid enhancer binding factors (4). Noncanonical Wnts (e.g., Wnt5a) activate alternate downstream pathways. Both Wnt pathways are implicated in cancers, with effects on proliferation, stemness, and immune evasion (5, 6).

Targeting downstream Wnt signaling therapeutically has proven challenging. Biologicals that inhibit upstream components of the pathway (such as blocking Wnt binding to Frizzled receptors), have showed limited efficacy and had dose-limiting toxicities in the clinic (7). However, these trials were conducted in broad patient populations, often in combination with chemotherapy, with no attempt to prospectively identify patients with Wnt ligand–dependent cancers who may benefit most.

Recently, small-molecule inhibitors have been developed targeting the membrane-bound *o*-acyl transferase Porcupine, which is essential for palmitoylation and secretion of active canonical or noncanonical Wnt ligands (8, 9). Although tumors with downstream Wnt pathway aberrations, such as in APC or β-catenin (encoded by *CTNNB1*), are unlikely to be susceptible to Porcupine inhibition, these inhibitors could be effective where upstream Wnt pathway aberrations drive the pathway. The Wnt negative regulator RNF43, a transmembrane E3 ligase, targets Frizzled receptors for lysosomal destruction (10). Somatic mutations in *RNF43* occur 9%–10% of colorectal (∼4% in microsatellite stable disease) and approximately 7% of pancreatic cancers (11, 12). Upstream Wnt signaling is also enhanced by extracellular R-spondins (RSPO), which prevent RNF43 binding to LRP/Frizzled and promote membrane clearance of RNF43 (13). Activating *RSPO2* or *RSPO3* fusions occur in 2%–10% of colorectal cancers (14). Upstream aberrations (*RNF43*-mutants and *RSPO*-fusions) appear mutually exclusive of each other and of downstream aberrations in *APC* or *CTNNB1* (14, 15). Here we report the discovery of RXC004, a potent and selective Porcupine inhibitor, its characterization in genetically defined colorectal and pancreatic cancer models, and its effects on Wnt-dependent immune suppression.

## Materials and Methods

### Animals

Unless specified, all experimental animals were sourced from Charles River. Protocols, including prespecified termination conditions associated with animal welfare and/or study endpoints, were approved by local institutional ethical review committee.

### Wnt Palmitoylation Assay

Palmitoylation of Wnt was performed by the Burris group, under a fee-for-service agreement, and was detected as described in Galli and colleagues (16). Briefly, HEK293T cells were transfected with GFP-Wnt1-Fc constructs, followed by overnight incubation with alkyne palmitate (Alk-C16) in the presence of DMSO or 100 nmol/L RXC004. Cells were lysed and A/G agarose beads used to pull down GFP-Wnt1-Fc, which was then subjected to click chemistry to covalently attach biotin azide to any attached alkyne palmitate. Following SDS-PAGE and Western blotting, palmitoylation of Wnt was detected using Streptavidin IR 800 dye. GFP detection served as a transfection and/or loading control.

### Wnt Secretion Assay

L-Wnt5a cells (L cells overexpressing Wnt5a; ATCC CRL-2814) were treated with RXC004 (300 nmol/L) or DMSO for 48 hours. Wnt5a secreted into media was detected by Western blotting using anti-Wnt5a antibody (Cell Signaling Technology #2530).

### Wnt Pathway Inhibition Analyses

Reporter Assay: Media from L-Wnt3a cells (L cells overexpressing Wnt3a; ATCC CRL-2647) treated with a concentration-series of RXC004, was incubated with the LEADING LIGHT Wnt Reporter Cell Line (Enzo life sciences; ENZ-61002-0001). Luciferase was detected using the ONE-Glo Luciferase Assay System (Promega E6120), data were normalized to DMSO-treated controls and IC_50_ values determined. Recombinant Wnt3a (Bio-Techne #1324-WN-010) was used at 1 μg/mL. β-catenin expression: L-Wnt3a cells were treated with RXC004 (100 nmol/L) or DMSO (0.1%) for 24 hours. Cellular β-catenin protein levels were quantified by staining with anti-β-catenin (AlexaFluor488, BD 562505) and assessment using an Agilent Novocyte-3000 flow cytometer.

### Caspase Analysis

Cells were treated with RXC004 (100 nmol/L) or DMSO (0.02%) for 7 days. Caspase 3/7 activity was measured using Caspase-Glo 3/7 Assay System (Promega #G8091) and data were plotted as a ratio relative to cell number as quantified using ATPlite 1-Step Reagent (Perkin Elmer #6016731).

### *In Vitro/In Vivo* Drug Metabolism and Pharmacokinetics

Aqueous solubility: A total of 1 mL of buffer was added to 1 mg of compound and incubated for 24 hours (Bioshake iQ, 650 rpm, 25°C). Following filtration under positive pressure, compound concentration in solution was assessed by high-performance liquid chromatography coupled to an ultraviolet detector (LC-UV).

Lipophilicity (logD_7.4_): A total of 30 μL of a 20 mmol/L stock solution of RXC004 was added to a vial containing 1.5 mL of pH7.4 phosphate buffer saturated octanol and 1.5 mL of octanol-saturated pH7.4 phosphate buffer, shaken and centrifuged to ensure phase separation. logD_7.4_ was calculated as the logarithm of the ratio of the concentration of RXC004 (measured by LC-UV in octanol vs. buffer phase).

pK_a_s were determined using the fast UV (spectrometric) technique. Samples were titrated under methanol-water mixture between pH2.0 and pH12.0 at 30–15 μmol/L at 25°C. The ionic strength was 0.192 mol/L (KCl). Aqueous pK_a_s were determined per Yasuda-Shedlovsky (17).

Metabolic stability: liver microsomes and hepatocytes from human, mouse (CD1), rat (Sprague Dawley) and dog (Beagle) were obtained from Invitrogen. RXC004 (1 μmol/L) was incubated at 37°C with microsomes (0.5 mg · mL^−1^) or hepatocytes (0.5 × 10^6^ cells · mL^−1^). Aliquots were removed at 0–45 minutes and RXC004 concentrations determined by LC/MS-MS analysis. Half-life (T_1/2_) and intrinsic clearance (CL_int_) were calculated as described in ref. 18.

Plasma protein binding: Mouse, rat, dog, and human plasmas were obtained from TCS Biosciences Ltd and Sera Laboratories Ltd. A total of 20 μmol/L RXC004 in 10% plasma diluted with phosphate buffer was dialyzed against phosphate buffer for 18 hours at 37°C. The concentration of RXC004 each side of the dialysis plate was determined by LC/MS-MS. Results were corrected to 100% plasma.

Cytochrome P450 (CYP) assays were performed using human liver microsomes incubated with P450 specific substrates as recommended by the FDA (19) at a concentration equivalent to *K*_m_ in the presence of 0.03–30 μmol/L RXC004, or solvent control. Concentration of the metabolites was analyzed by LC/MS-MS.

Permeability of RXC004 was assessed in MDR1-MDCKII (Netherlands Cancer Institute) and Caco-2 cells (Merck Life Sciences). RXC004 (10 μmol/L) was added to either apical or basolateral chamber for 2 hours at 37°C, then an aliquot from the opposite side of the monolayer was removed and quantified by LC/MS-MS. Apparent permeability (*P*_app_) was calculated according to standard practice (20).

*In vivo* pharmacokinetics: RXC004 was dosed to male Sprague Dawley rats, male CD1 mice or dog (beagle) by intravenous and oral routes. Bioanalysis of blood and plasma samples was performed by LC/MS-MS using a Waters TQS or TQMS mass spectrometer. Pharmacokinetic information was derived using WinNonlin software (Certara).

### *In Vivo* Toxicity in Mice

Groups of 9–10 SCID-Beige mice were dosed at Translational Drug Discovery with vehicle, 1.5 mg/kg or 5 mg/kg RXC004 orally twice daily, or 5 mg/kg RXC004 orally once daily, for 28 days. Formulation: 5% DMSO, 95% hydroxyl propyl beta cyclodextrin (25% w/v). Formalin-fixed, paraffin-embedded (FFPE) samples from the small intestine were assessed by hematoxylin and eosin (H&E) and/or Ki67 staining at Histologix.

### *In Vitro* Profiling Across Genetically Defined Cancer Cell Lines

Cells were treated with a concentration-series of RXC004 for 5 days and proliferation measured using ATPLite 1-Step Reagent (Perkin Elmer #6016731). Data were normalized to DMSO-treated controls and IC_50_ values determined. Colorectal cell lines used were: WiDR (*APC*-E853*/T1556fs*3), HCT116 (β-cateninΔSer45; *RNF43*-R117fs*41), JVE-109 (*RNF43*-V211fs*47/G659fs*41), SNU-1411 (*PRPTK-RSPO*-3 fusion). Pancreatic cell lines were: CAPAN-2 (*RNF43*-R330fs*89), AsPC1 (*RNF43*-S720*), and HPAF-II (*RNF43*-E174*). Cell lines were from ATCC, except for SNU-1411 (Korean Cell Line Bank) and cultured for no more than 20 passages. Cells were routinely tested for *Mycoplasma* by the MycoAlert test (Lonza) and authenticated using short tandem repeat fingerprinting.

The expression of nine potential Wnt-responsive genes was screened across cell lines by treatment with RXC004 (10 nmol/L) or DMSO (0.1%) for 3 days. RNA was extracted (Aurum RNA isolation kit, Bio-Rad #372-6820) and gene expression assessed by Taqman qPCR relative to appropriate housekeeping genes on the Bio-Rad CFX real-time system.

For concentration–response effects on gene expression, Single-Shot Cell Lysis kit (Bio-Rad #172-5080) was used for RNA extraction, data were normalized to DMSO-treated controls, and IC_50_ values determined. For cell-cycle analysis, the cancer cell line panel was treated with RXC004 (100 nmol/L) or DMSO (0.1%) for 3 days. Cell-cycle profiles were determined by staining with propidium iodide (Thermo Fisher Scientific #P3566) and phospho-Ser10-Histone-H3 (AlexaFluor-647, BioLegend #650806), and quantified using an Agilent Novocyte-3000 flow cytometer. For *in vitro* growth curve analysis, HPAF-II and SNU-1411 cells were seeded in 96-well plates at 2,000 cells/well and imaged on the IncuCyte (Sartorius) at 10× magnification with 4 fields of view per well. Total cell object count over time was calculated using the Adherent Cell-by-Cell analysis module.

### *In Vivo* Xenograft Models

The *in vivo* pilot studies were performed at Axis Bioservices. HPAF-II (5 × 10^6^ cells; athymic nude mice), AsPC1 (3 × 10^6^ cells; athymic nude mice), and SNU-1411 (1 × 10^7^ cells; NOD-SCID mice) were implanted bilaterally, subcutaneously, whereas HCT116 (3 × 10^6^ cells; athymic nude mice) were implanted in a single flank. Animals were randomized into the indicated groups and treatment initiated once tumor volumes reached approximately 100–150 mm^3^. Dosing was either 1.5 mg/kg twice daily RXC004 for 7–13 days then once daily for the remainder of study (up to 29 days), or 28 days 1.5 mg/kg twice daily RXC004 for HCT116. In these and all other animal studies, unless otherwise specified, RXC004 was dosed orally in 0.5% carboxymethylcellulose + 0.1% Tween-80. For AsPC1, an ascites model, end of study tumor weight (mg) was measured. A total of 12 hours after the final dose, RNA was extracted [GenElute RNA isolation kit (#RTN70, Sigma)] from snap frozen tumor for qRT-PCR expression analysis of Axin2, c-Myc, RNF43, MUC4, and MUC5AC relative to appropriate housekeepers.

To explore dose–response effects *in vivo*, SNU-1411 (1 × 10^7^ cells) were implanted into NOD-SCID mice subcutaneously at Axis Bioservices. Animals were randomized into the indicated groups and oral dosing of RXC004 was initiated when tumors reached approximately 200 mm^3^. Tumor volume measurements were taken at day 19, *n* ≥9 per group. Pharmacokinetic/pharmacodynamic analysis in SNU1411/NOD-SCID mice was performed in a separate study at Axis Biosciences, with tissue and plasma extracted for assessments after 7 days of RXC004 dosing. Tumor was divided into two sections; one section snap frozen and the other processed by FFPE. RXC004 levels were determined in snap frozen tumor and blood samples by LC/MS-MS. Expression analysis of the indicated genes was performed on snap frozen tumor as described above. Serial FFPE tumor sections were stained with H&E, anti-Ki67 (Abcam #Ab15580) or the dual mucin stain Alcian-blue/periodic acid shift (AB-PAS) at Histologix. IHC images were quantified using HALO software and modules (Indica Labs). The AB-PAS images were analyzed with the area quantification module on tumor area. A classifier was trained to detect differentiated and nondifferentiated tumor areas in RXC004-treated tumors using the AB-PAS–stained slides. Image registration was used to assign annotations from AB-PAS images to Ki67 images. The Ki67 images were analyzed with the multiplex IHC module and number of Ki67-positive cells were determined per mm^2^ of total or differentiated tumor area.

The reimplantation study was performed at Sygnature Discovery. SNU-1411–implanted NOD-SCID mice were dosed orally with RXC004 (5 mg/kg, once daily) once tumors reached an average size of 350 mm^3^. After 9 days treatment, tumors were resected and equivalent viable tumor fragments from each tumor were reimplanted into separate naïve NOD-SCID mice, tumor volume was assessed at various subsequent timepoints.

B16F10/C57BL/6 syngeneic model was performed at Axis Bioservices. Mouse B16F10 cells (2 × 10^5^) were subcutaneously implanted in flanks of the immunocompetent male C57BL/6 mice. Treatment was initiated at day 3, once tumors were palpable with RXC004 being dosed orally as indicated. Anti-programmed cell death protein-1 (PD-1; RMP1-14, BioXcell) or isotype control was dosed at 100 μg per dose intraperitoneal, twice weekly. Anti-CTLA-4 antibody (BioXCell BP0131) or isotype control was dosed intraperitoneally every 3 days at 100 μg per dose. Separate experiments were performed for tumor volume and flow cytometry analysis. Tumor volume was assessed in each animal and/or group until prespecified termination conditions and/or study endpoint were reached. For flow cytometry, tumors were resected at day 19, 2 hours after final dose, weighed and digested into a single-cell suspension using collagenase (Liberase, Roche) and DNAase and passed through a strainer prior to flow cytometry analysis on FACS Canto II apparatus using Flow JO software. Single and fluorescence minus one stains were used for compensation setup and gating controls respectively. A myeloid analysis panel included Zombie viability dye (BioLegend #423106), CD11b (BioLegend #101228), CD45, Ly6C, and Ly6G (BD Biosciences #563891, #562727, #551460). Myeloid myeloid-derived suppressor cells (MDSC) were gated as CD45^+^CD11b^+^ Ly6G-Ly6C^high^ and polymorphonuclear MDSCs were gated as CD45^+^CD11b^+^ Ly6G^+^Ly6C^med^ (21). For gene expression analysis, tumors were snap frozen, total RNA was extracted and mRNA expression levels for various genes analyzed using the Nanostring nCounter Mouse PanCancer IO360 Codeset.

B16F10/SCID-Beige model was performed at Axis Bioservices. Mouse B16F10 cells (2 × 10^5^) were subcutaneously implanted in flanks of the immunocompromised male SCID-Beige mice. Treatment with RXC004 (5 mg/kg once daily) was initiated at day 3, once the tumors were palpable, and tumor volume assessed over time.

CT26/BALB/c syngeneic model was performed at ProQuinase GmbH. Mouse CT26 cells (5 × 10^5^) were subcutaneously implanted in the flanks of the immunocompetent female BALB/c mice. Treatment was initiated once tumors reached approximately 50 mm^3^. RXC004 was dosed orally at 1.5 or 5 mg/kg (once daily). Anti-PD-1 (BioXCell) or isotype control was dosed every 3 days at 10 mg/kg i.p. Tumor volume was measured over time. For immunophenotyping the tumor infiltrate, tumors from a separate study were resected at day 14, weighed, digested, and stained for flow cytometry analysis by Beckton Dickenson LSR Fortessa. T-cell analysis panel included viability dye, CD45, CD3, CD4, CD8, CD25, and FOXP3.

### *In Vitro* Glucose Uptake

SNU-1411, HPAF-II, and HCT116 cells were treated *in vitro* for 48 hours with RXC004 (100 nmol/L), Latrunculin A (Abcam #ab144290; 500 nmol/L), or DMSO prior to analysis using the Glucose Uptake Glo Assay Kit (Promega #J1341). The ATPlite assay was used to control for cell viability, confirming RXC004 has no effect on proliferation at this 48 hours timepoint.

### *In Vivo* ^18^fluorodeoxyglucose-PET

Experiment was performed at Medicines Discovery Catapult. SNU-1411 (1 × 10^7^ cells; NOD-SCID mice) or HPAF-II (1 × 10^7^ cells; NOD-SCID mice) were implanted subcutaneously and treatment with RXC004 (5 mg/kg once daily) or vehicle commenced when tumors reached approximately 200 mm^3^. Mice were imaged prior to dosing, day 3 and day 7 after dosing. A 10 MBq injection of ^18^FDG (PETNET), with a 45-minute washout was followed by a 20-minute PET scan using a Siemens Inveon PET scanner. Tissues removed on day 7 for biodistribution analysis were assessed using a PerkinElmer Wallac Wizard Gamma counter.

### *In Vitro* Human Peripheral Blood Mononuclear Cell Assays

HPAF-II cells were incubated for 3 days with RXC004 (100 nmol/L) or DMSO control, washed and detached using Accutase and equal number of viable cells (as assessed by Trypan Blue exclusion) per treatment were reseeded in 96-well plates. Frozen human peripheral blood mononuclear cells (PBMC), originally collected by density gradient separation using Histopaque from four healthy donors, were thawed and cultured in complete media. After approximately 40 hours, nonadherent PBMCs were added to coculture with the HPAF-II cells, at a PBMC:HPAF-II ratio of 5:1. T-cell proliferation and cytokine production was stimulated using Immunocult CD3/CD28-tetramer (Stemcell Technologies #10971). For monoculture nonadherent PBMC experiments, T cells were stimulated using Immunocult in the presence or absence of recombinant Wnt3a (Biotechne #5036-WN-010) and/or recombinant Wnt5a (Biotechne #645-WN-010), both at 100 ng/mL. After 5 days, supernatants from monoculture or coculture experiments were removed for analysis, whereas suspension cells were fixed, stained with anti-Ki67 (BD #563462), anti-CD4 (BioLegend #317420), and anti-CD8 (BD #564116) antibodies, before flow cytometry was performed on a Novocyte flow cytometer. Cytokines and chemokines in the supernatant were quantified using a 30-plex panel on Magpix (Thermo Fisher Scientific #LHC6003M) or ELISA for IFNγ (R&D Systems # DY285B-05).

### Statistical Analysis

Unless stated otherwise, drug metabolism and pharmacokinetic (DMPK) data analysis was performed using Excel (Microsoft). All other statistical analyses were performed using Prism software (GraphPad Prism). For each analysis, residual plots were examined and where necessary a log transformation was applied prior to analysis. Where indicated, IC_50_ values were determined using 4-parameter curve fitting within Prism software.

### Data Availability Statement

The data generated in this study are available upon request from the corresponding author.

## Results

### Structure and *In Vitro* Characterization of RXC004

**FIGURE 1:**
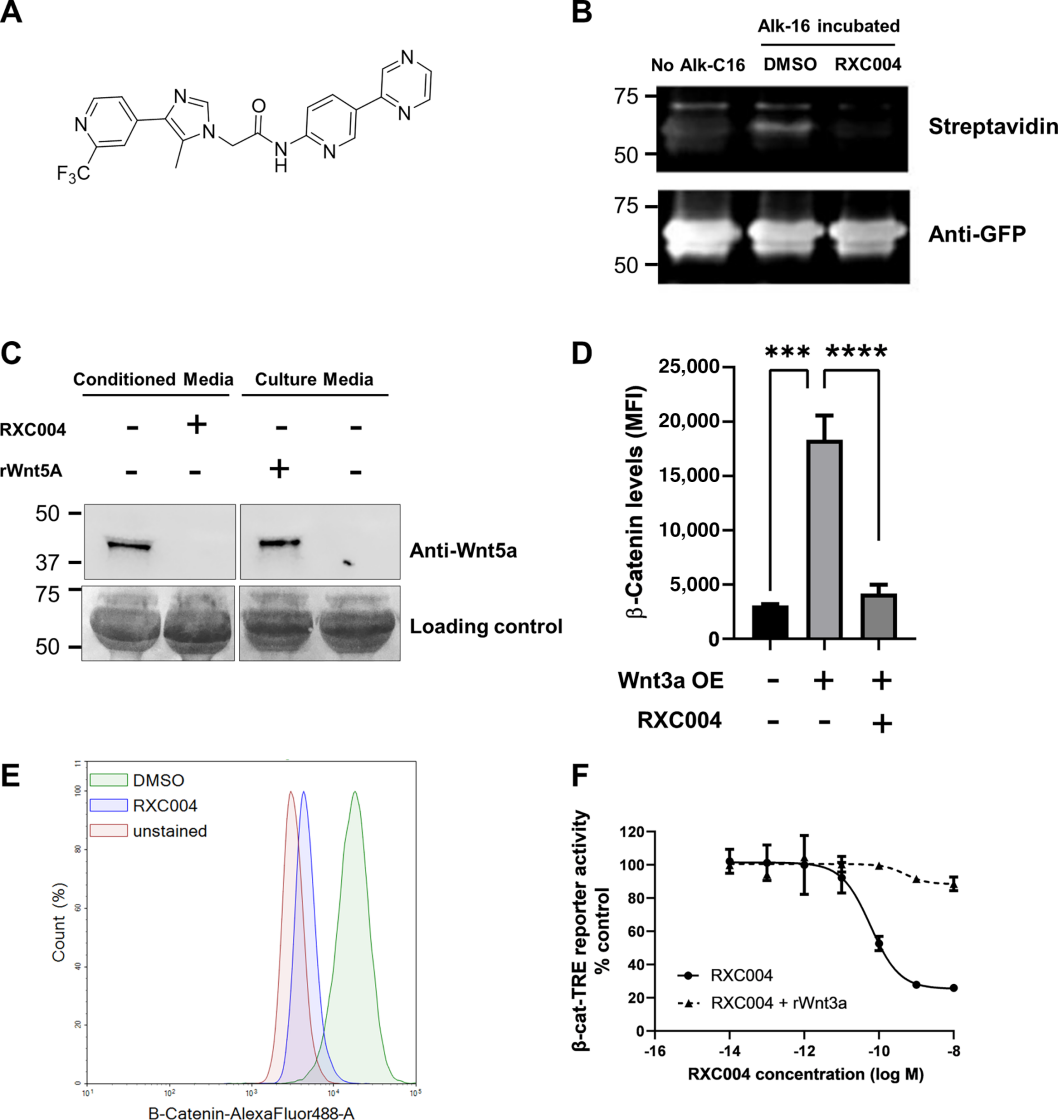
RXC004 structure and *in vitro* compound characterization. **A,** Structure of RXC004. **B,** RXC004 (100 nmol/L) inhibits palymitoylation of GFP-tagged Wnt1-Fc transfected into HEK293T cells and incubated with alkyne palmitate (Alk-C16). Palymitoylation was quantified by click chemistry attachment of biotin, following immunoprecipitation of GFP-Wnt1-Fc, and Western blotting using Streptavidin IR-800, which binds to biotin. Anti-GFP was used as loading/transfection control. **C,** RXC004 (300 nmol/L) inhibits secretion of Wnt5a from L cells into the conditioned media, as detected using Western blotting with anti-Wnt5a. Recombinant Wnt5a (rWnt5a) added to culture media demonstrates antibody specificity. **D,** Wnt3a overexpression (OE) in L cells leads to increased β-catenin protein levels, indicative of canonical Wnt pathway activation, as detected by flow cytometry. Treatment with 100 nmol/L RXC004 reduces β-catenin protein levels in L-Wnt3a cells back to baseline. Data are mean + SEM of *n* = 3 experiments, statistical comparison by one-way ANOVA, *P* < 0.001 denoted by “***”, *P* < 0.0001 denoted by “****”. **E,** Representative flow cytometry plot showing RXC004 (100 nmol/L) effects on L-Wnt3a cell β-catenin protein levels versus DMSO controls. **F,** Concentration-dependent inhibition of functional Wnt production from mouse L-Wnt3a cells by RXC004, as detected by β-catenin transcriptional response element (TRE) luciferase reporter activity. Transcription is rescued by addition of recombinant Wnt3a (rWnt3a). Data shown are means ± SD, *n* = 6.

A rational design approach was used to identify potent novel Porcupine inhibitors. Routine *in vitro* screening allowed for the elucidation of structure activity relationships which ultimately resulted in the discovery of RXC004: 2-[5-methyl-4-[2-(trifluoromethyl)-4-pyridyl]imidazol-1-yl]-N-(5-pyrazin-2-yl-2-pyridyl)acetamide; structure shown in Fig. 1A.

RXC004 inhibited palmitoylation of Wnt, indicating direct inhibition of the membrane-bound *o*-acyl transferase Porcupine (Fig. 1B). Consistent with this, RXC004 inhibited Wnt5a secretion from L-Wnt5a cells (L-cell stably transfected to express Wnt5a) into the media (Fig. 1C), and reduced levels of β-catenin in L-Wnt3a cells (L-cell stably transfected to secrete Wnt3a ligand; Fig. 1D and E). Moreover, RXC004 treatment of L-Wnt3a cells reduced the ability of their conditioned media to activate a β-catenin responsive luciferase reporter in a concentration-dependent manner, with an IC_50_ of 64 pmol/L. Luciferase activity was restored by the addition of recombinant Wnt3a, demonstrating that RXC004 had no effect on downstream Wnt signaling (Fig. 1F).

### In Vitro and In Vivo DMPK

The physicochemical profile of RXC004 is shown in Table 1. The solubility of RXC004 was 56 μmol/L in pH 7.4 buffer, with a logD (pH7.4) of 2.4. pK_a_s were 3.8 (most basic) and 11.2 (most acidic), meaning that the predominant species at pH 7.4 is the neutral, nonionized form of the molecule. The average percentage free of RXC004 in plasma across species ranged from 2.5% to 7.5%. Microsomal CL_int_ values ranged from 3.9 to 31.6 μL · minute^−1^ · mg^−1^, with mouse having the lowest and dog the highest predicted clearances. Hepatocyte CL_int_ values ranged from < 3 to 12.5 μL · minute^−1^ · 10^6^ cells, with dog displaying moderate clearance while rodents and humans display low clearance. RXC004 has good intrinsic permeability, showing some evidence of efflux in MDR1-MDCKII cells but not in Caco-2 cells.

**TABLE 1:** DMPK profiling of RXC004

Parameter	Value
**Molecular weight (Da)**	439.4
**Measured log D_7.4_**	2.44
**pK_a_**	3.84, 11.2
**Solubility at pH 7.4 (μmol/L)**	56.0
**Plasma protein binding (% free)**	
Rat	5.0
Mouse	7.5
Dog	2.5
Human	4.2
**Permeability**	
Caco-2 (P_app_ × 10^6^ cm/s)	P_appA-B_ 23.6 P_appB-A_ 21.8
MDCK (P_app_ × 10^6^ cm/s)	P_appA-B_ 15.0 P_appB-A_ 51.9
**Hepatocyte stability: CL_int_ (μL minute^−^^1^ · 10^6^ cells^−^^1^)**	
Rat	5.3
Mouse	<3.0
Dog	12.5
Human	<3.0
**Microsomal stability: CL_int_ (μL minute^−^^1^ · mg^−^^1^)**	
Rat	7.8
Mouse	3.9
Dog	31.6
Human	6.0
**Cytochrome P450 IC_50_ (μmol/L)**	
(CYP1A2, CYP2C9, CYP2C19, CYP2D6, CYP3A4)	All >25

NOTE: P_app_, effective permeability; P_appB-A_, permeability in basolateral to apical direction; P_appA-B_, permeability in apical to basolateral direction; CL_int_, intrinsic clearance.

RXC004 displays *in vitro* absorption, distribution, metabolism, and excretion properties which are predictive of the low metabolic clearance and good oral bioavailability, as successfully observed *in vivo* in mouse, rat, and dog (Supplementary Table S1). Volume of distribution *in vivo* was low, resulting in a short *in vivo* half-life in preclinical species (Supplementary Table S1). Low concentrations of an acidic metabolite and hydroxylated versions of RXC004 have been identified from microsomes and hepatocytes of mouse, rat, dog, and human. In recombinant CYP enzyme assays, RXC004 was exclusively metabolized by CYP3A4. RXC004 does not inhibit any of the five major CYPs at concentrations of biological relevance (Table 1).

### *In Vitro* Profiling of RXC004 Across Genetically Defined Human Cancer Cell Lines

**FIGURE 2:**
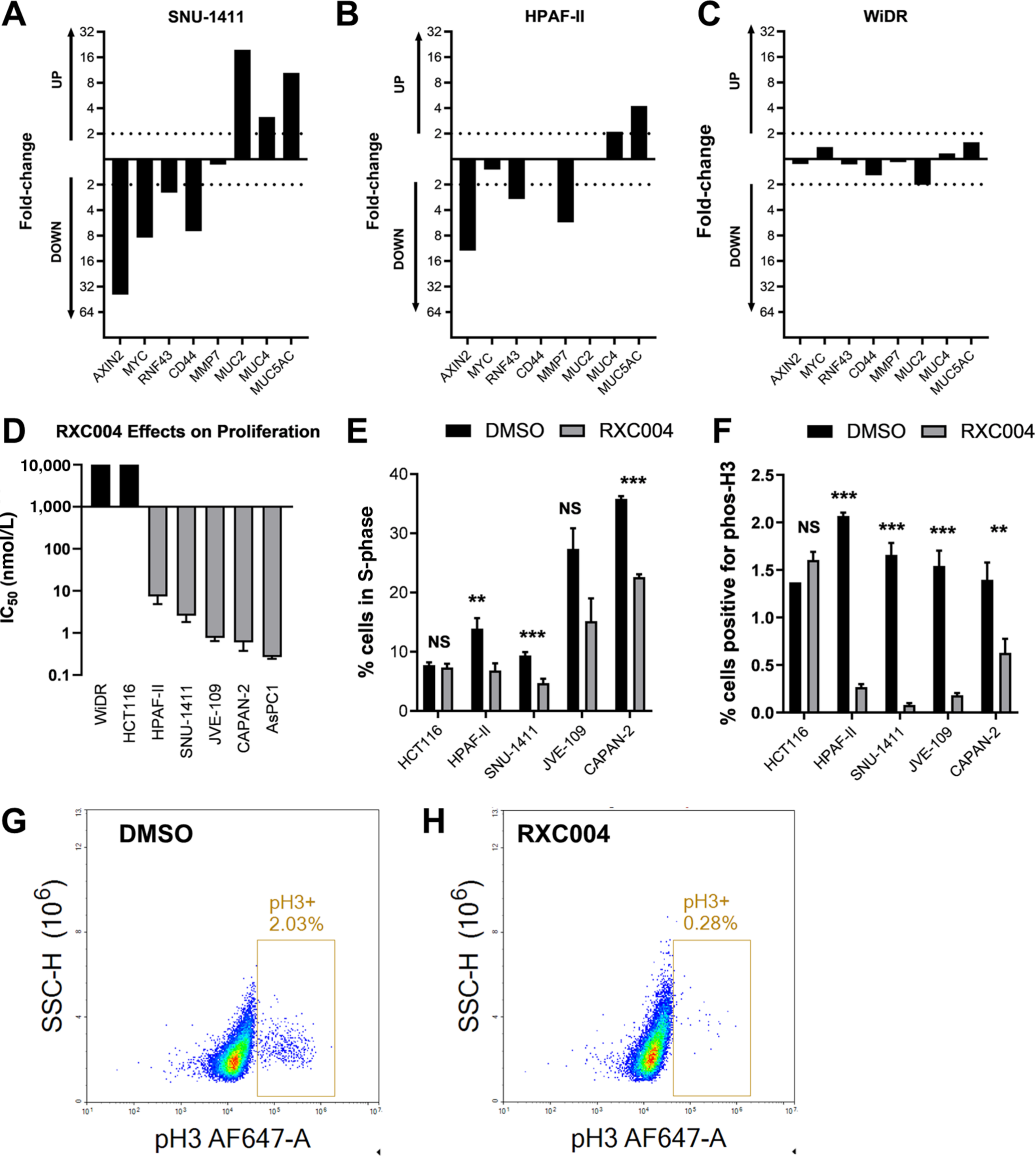
RXC004 profiling in genetically defined human pancreatic and colorectal cancer cell lines. RXC004 (10 nmol/L) effects on gene expression in predicted Wnt ligand–dependent SNU-1411 (**A**) and HPAF-II (**B**) cells, or Wnt ligand–independent WiDR (**C**) cells. Data presented are representative examples, showing fold differential mRNA expression versus DMSO controls. **D,** Concentration-dependent effects of RXC004 on proliferation in various cancer cell lines. Data plotted are IC_50_ means and SEM of *n* ≥ 3 experiments. Cell-cycle arrest in Wnt ligand–dependent cell lines by RXC004 (100 nmol/L) as assessed by flow cytometry with propidium iodide (**E**) or phospho-Ser10 histone H3 (**F**). Data are mean + SEM, *n* ≥3 except for HCT116, (*n* = 1–4), *P* < 0.01 denoted by “**”, *P* < 0.001 denoted by “***” (unpaired *t* test). Representative flow cytometry plots for phospho-Ser10 histone H3 staining of HPAF-II cells treated with DMSO (**G**) or 100 nmol/L RXC004 (**H**); box denotes the positive population with the percentage positive cells indicated.

The effect of RXC004 on expression of Wnt-responsive genes and cell proliferation was evaluated across a panel of colorectal and pancreatic cancer cell lines. These included cell lines with upstream Wnt pathway aberrations such as loss-of-function (LoF) mutations in *RNF43* (HPAF-II, CAPAN-2, JVE-109, and AsPC1) or gene fusions in *RSPO3* (SNU-1411), and those with downstream Wnt pathway aberrations such as LoF mutations in *APC* (WiDR) or gain-of-function mutations in *CTNNB1* (HCT116; ref. 22). As predicted, Porcupine inhibition by RXC004 downregulated mRNA expression of Wnt pathway targets Axin2, RNF43 (both involved in the negative-feedback mechanism of the Wnt pathway), c-Myc, CD44, and MMP7 in cell lines with *RNF43* LoF mutations and *RSPO3* fusions (Fig. 2A and B; Supplementary Fig. S1A–S1C). In addition, RXC004 caused a marked increase in mucin mRNA, a marker of epithelial cell differentiation (Fig. 2A and B; Supplementary Fig. S1A–S1C). Minimal effects of RXC004 on Wnt pathway target expression were observed in WiDR and HCT116, cell lines where Wnt signaling is activated by downstream mutations (Fig. 2C; Supplementary Fig. S1D).

RXC004 inhibited proliferation of cell lines with *RNF43* mutations and *RSPO3* fusions in a concentration-dependent manner but had no effect on cell lines with mutations in downstream Wnt signaling (Fig. 2D). Effects of RXC004 on proliferation were mirrored by a concentration-dependent downregulation of c-Myc mRNA (Supplementary Fig. S1E). Further analysis demonstrated that RXC004 reduced the proportion of cells in S-phase, and strongly inhibited expression of the mitosis marker phospho-histone-H3 in cells with upstream aberrations in Wnt pathway components (Fig. 2E–H), indicative of cell-cycle arrest. RXC004 did not induce cell death, as confirmed by lack of significant effect on Caspase 3/7 activity in SNU-1411 and HPAF-II cell lines (Supplementary Fig. S1F and S1G), and as evidenced by the delayed inhibition of cell growth by IncuCyte growth curve analysis (Supplementary Fig. S1H and S1I).

### Effects of RXC004 on Tumor Growth *In Vivo*

*In vivo* xenografts models of SNU-1411, AsPC1, HPAF-II, and HCT116 cell lines were used to test a single dose of RXC004 (1.5 mg/kg twice daily). A reduction in tumor growth, and inhibition of Wnt-responsive gene expression including c-Myc, was observed in the Wnt ligand–dependent SNU-1411, AsPC1, and HPAF-II models (Supplementary Fig. S2). Moreover, mRNA for MUC5AC and MUC4 were upregulated, consistent with increased epithelial differentiation. No effect of RXC004 on tumor growth was seen in the Wnt ligand–independent HCT116 xenograft model (Supplementary Fig. S2P).

**FIGURE 3:**
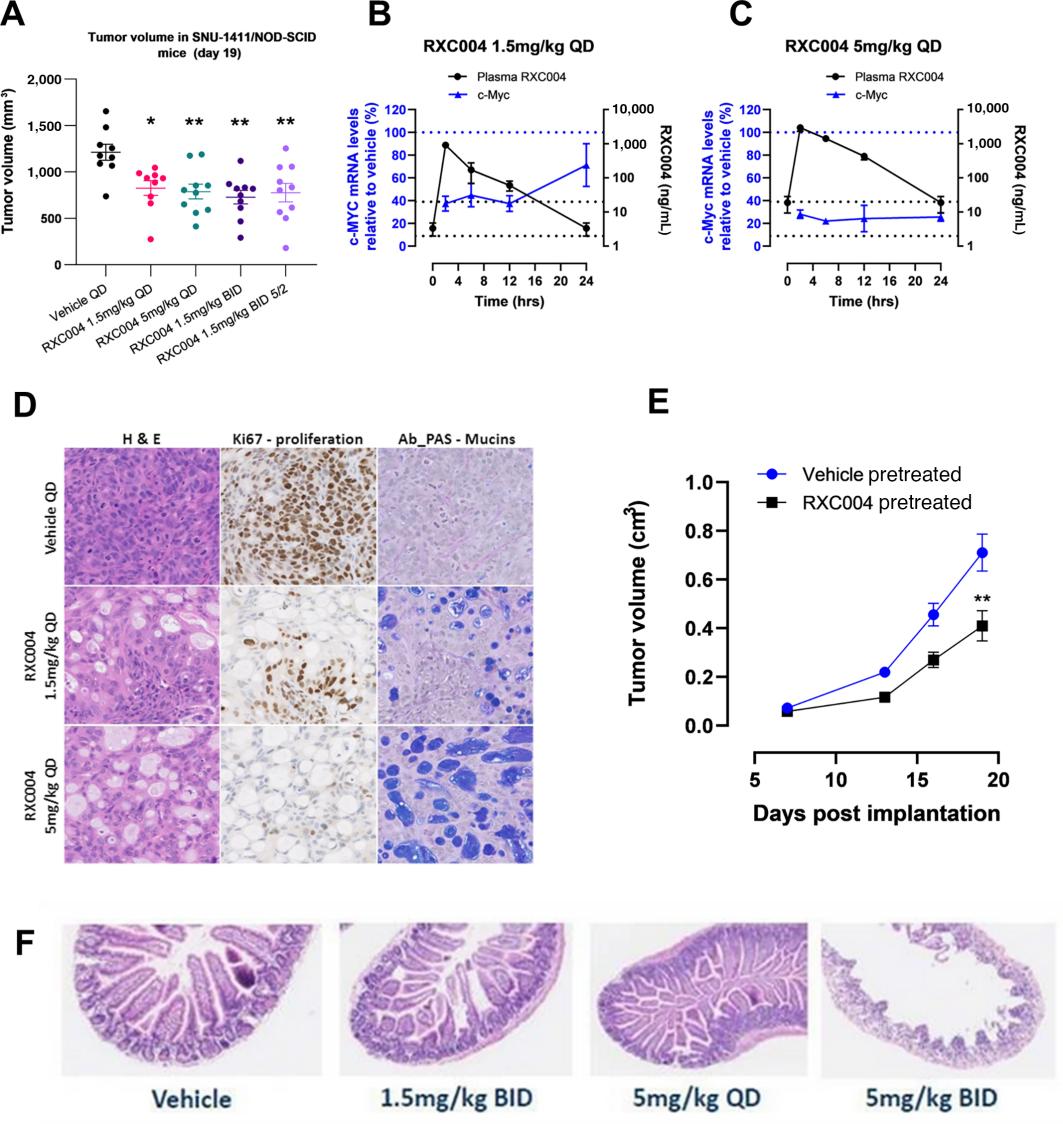
Effects of RXC004 in SNU-1411/NOD-SCID xenografts. **A,** Effect of various RXC004 dosing regimens on tumor volume in SNU-1411–implanted NOD-SCID mice. QD, once daily; BID, twice daily; 5/2, 5 days dosing interspersed with 2 days without dosing. Mean ± SEM are represented alongside individual datapoints. Total plasma concentration of RXC004 over time is shown in black, following final doses of 1.5 mg/kg once daily (**B**) or 5 mg/kg once daily (**C**). c-Myc mRNA expression in SNU-1411 tumors over time (relative to control) is shown in blue, following final treatment with RXC004 at the indicated doses. Horizontal black dotted lines represent 2 or 20 ng/mL RXC004 (equivalent to free IC_50_ or 10× IC_50_, respectively). Blue dotted line represents no inhibition of c-Myc expression compared to control. *n* = 4. Data are mean ± SEM. **D,** RXC004 (1.5 or 5 mg/kg once daily) effect on H&E, Ki67 staining, and mucin expression measured by AB-PAS. 20× magnification. **E,** Tumors from SNU-1411/NOD-SCID mice treated with vehicle or 5 mg/kg once daily RXC004 were removed and reimplanted into naïve, untreated NOD-SCID hosts (*n* = 16 per group) and ensuing tumor volume measured up to 20 days postreimplantation. **F,** SCID-Beige mice treated for 28 consecutive days with vehicle, RXC004 1.5 mg/kg twice daily, 5 mg/kg once daily, or 5 mg/kg twice daily. Loss of villi architecture observed at 5 mg/kg twice daily only. Statistical comparison; ordinary one-way ANOVA (**A**) or unpaired *t* test (**E**). *P* < 0.05 denoted by “*,” *P* < 0.01 denoted by “**.”

Further efficacious dose schedules and pharmacokinetic/pharmacodynamic relationships were explored in the colorectal cancer *RSPO3*-fusion model SNU-1411. Significant reduction in tumor volume was observed at various doses of RXC004, including 1.5 and 5 mg/kg continuous once daily dosing, and 1.5 mg/kg twice daily dosing either continuous or on and a 5-day on/2-day off dosing schedule (Fig. 3A). In a separate 7-day study, pharmacokinetic analysis showed good dose proportionality of RXC004 levels in the plasma and tumor following oral administration of 1.5 or 5 mg/kg RXC004 once daily (Supplementary Fig. S3). Inhibition of c-Myc gene expression in tumor was observed at both doses (Fig. 3B and C), with more prolonged inhibition at the 5 mg/kg dose, which maintained free plasma concentrations above that required for approximately 10 × IC_50_ cover over 24 hours (Fig. 3C). Similar effects were observed on Axin2, RNF43, and CD44, while MUC4 and MUC5A mRNA increased in a dose-dependent manner (Supplementary Fig. S3). Consistent with this, increased tumor differentiation and increased mucin levels (as detected by Alcian-blue/PAS staining) were observed within tumor sections of RXC004-treated tumors, with intermediate effects at 1.5 mg/kg once daily and stronger effects at 5 mg/kg once daily (Fig. 3D; Supplementary Fig. S4A and S4B). Ki67-positive cells were reduced by RXC004 in terms of the total tumor area, and more significantly in areas of differentiated tumor (Supplementary Fig. S4C). Reimplantation of SNU-1411 tumors from RXC004-treated mice into naïve, untreated animals demonstrated a prolonged impact on tumor growth of the prior RXC004 treatment (5 mg/kg once daily) compared with vehicle pretreated controls (Fig. 3E).

To establish a therapeutic window with RXC004, mice were dosed for 28 consecutive days with RXC004 at 1.5 mg/kg twice daily or 5 mg/kg (once daily or twice daily). Disruption of villi architecture (villi clubbing, blunting, or fusion) was observed only in some animals dosed at 5 mg/kg twice daily. No effects were seen at either the 1.5 mg/kg twice daily or the 5 mg/kg once daily doses previously demonstrated to inhibit tumor growth and Wnt-responsive gene expression (Fig. 3F). Moreover, there was no effect on the Ki67 proliferation marker in sections of intestinal crypts at 1.5 mg/kg twice daily, but a clear decrease at 5 mg/kg twice daily which was associated with a loss of body weight (Supplementary Fig. S4D–S4G).

### RXC004 Suppression of Tumor Metabolism in Genetically Selected Cancer Models

**FIGURE 4:**
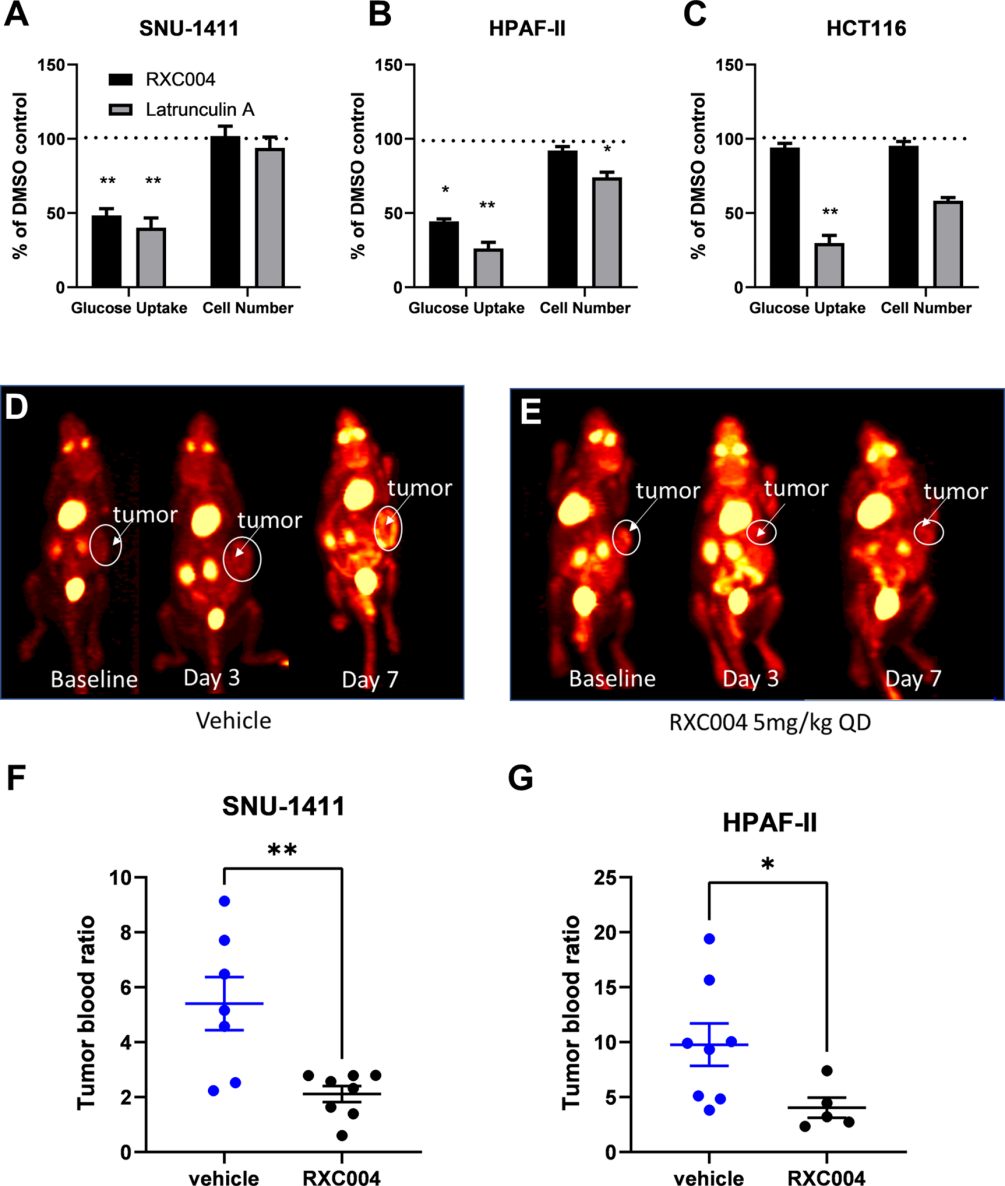
Effect of RXC004 on tumor cell metabolism. Glucose uptake following treatment with RXC004 (100 nmol/L; black bars) or Latrunculin A (500 nmol/L; gray bars) relative to DMSO controls in SNU-1411 (**A**), HPAF-II, (**B**) or HCT116 (**C**) cell lines. ATP-lite was used to control for effects on cell number, with RXC004 having no effect at this 48-hour timepoint. Data are mean _SEM, *n* = 3. Statistics by RM one-way ANOVA on raw data. SNU-1411/NOD-SCID mice treated with vehicle (**D**) or RXC004 at 5 mg/kg once daily (**E**) were imaged by ^18^FDG-PET at baseline, day 3 and day 7 after dose initiation, representative images with tumor region highlighted are shown for each treatment group. Day 7 tumor:blood biodistribution ratios for ^18^FDG in SNU-1411 (**F**) and HPAF-II (**G**) xenograft models treated with vehicle or RXC004 5 mg/kg once daily (*n* = 5–8), the mean ± SEM of each group is indicated alongside individual datapoints. Statistics by unpaired *t* test. *P* < 0.05 denoted by “*” and *P* < 0.01 denoted by “**.”

*In vitro*, RXC004 inhibited glucose uptake by over 50% in the Wnt ligand–dependent cell models SNU-1411 and HPAF-II cells, but had no effect in the Wnt ligand–independent HCT116 cell line (Fig. 4A–C). At this relatively early 48-hour timepoint, RXC004 had no effect on cell numbers as assessed by ATP-lite or by IncuCyte growth curve analysis (Supplementary Fig. S1H and S1I). The positive control Latrunculin A blocked glucose uptake in all three cell lines.

*In vivo* ^18^fluorodeoxyglucose-PET (^18^FDG-PET) imaging analysis in SNU-1411 xenograft models demonstrated a 31% decrease in maximum standardized uptake value in RXC004-treated tumors compared with vehicle controls at day 7. Moreover, a significant decrease in tumor:blood ratios was observed in both SNU-1411 and HPAF-II xenograft models following biodistribution analysis of the ^18^FDG tracer at end of study (7-day dosing; Fig. 4D–G).

### RXC004 Effects on Immune Evasion in the B16F10 “Cold” Tumor Model

**FIGURE 5:**
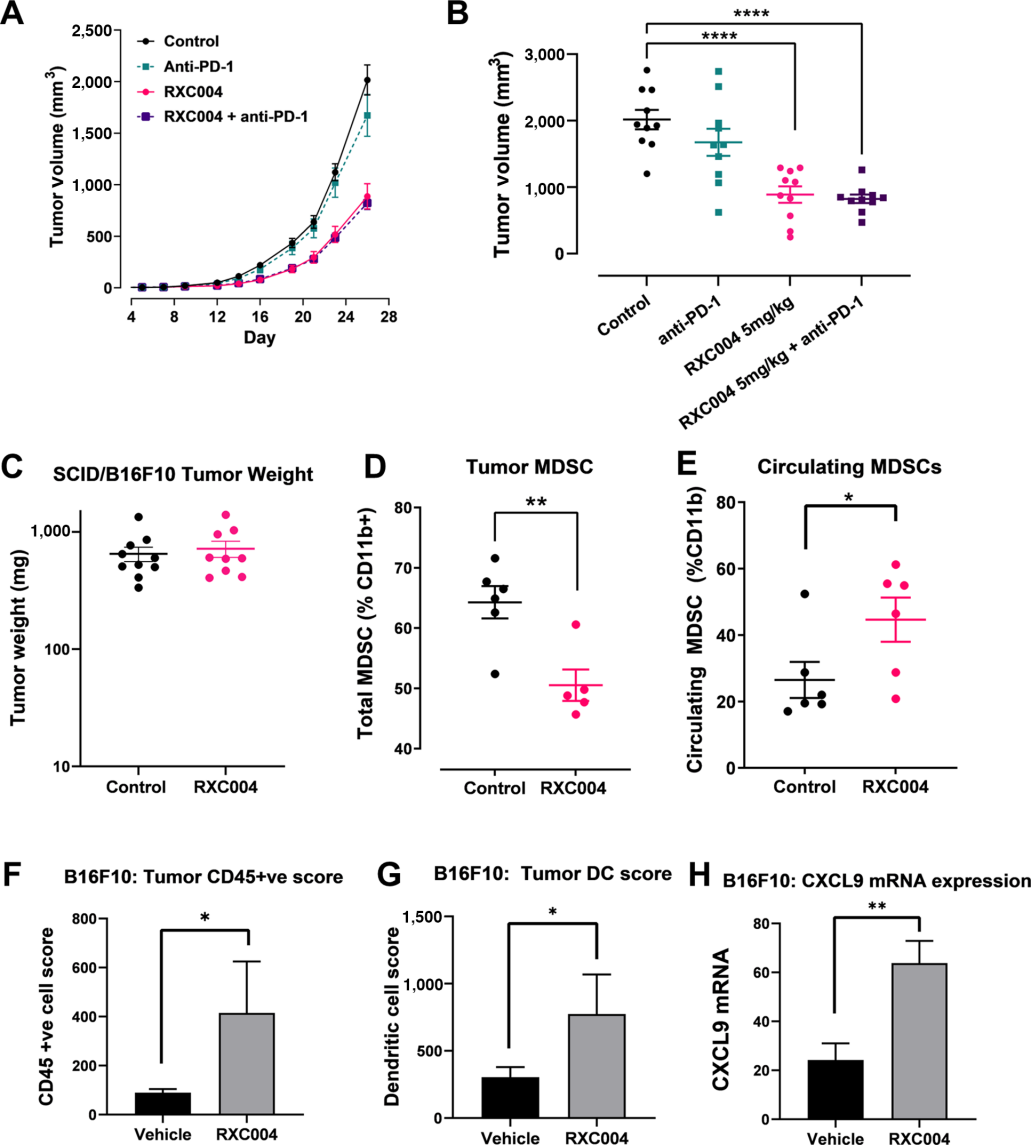
Effect of RXC004 in the B16F10 syngeneic model. **A,** Time course for vehicle, RXC004 (5 mg/kg once daily), anti-PD-1 antibody (100 μg per dose, twice a week), or RXC004 (5 mg/kg once daily) + anti-PD-1 combination treatment on tumor growth of B16F10 xenografts in C57BL/6 mice. *n* = 10 per group. Data are means ± SEM. **B,** Tumor volumes at day 26 in B16F10/C57BL/6 mice treated with vehicle, anti-PD-1 antibody (100 μg per dose, twice a week), or 5 mg/kg once daily RXC004 alone or in combination with anti-PD-1 antibody. Mean ± SEM is indicated alongside individual datapoints. **C,** Lack of inhibition of tumor growth in B16F10 explants in SCID-Beige immunocompromised mice treated with vehicle or RXC004 (5 mg/kg once daily for 21 days). Mean ± SEM is indicated alongside individual datapoints, *n* = 9–10 per group. Tumor-infiltrating MDSCs (**D**) or circulating MDSCs (**E**) in B16F10/C57BL/6 mice at day 19 of treatment with vehicle or RXC004 at 5 mg/kg once daily. Mean ± SEM is indicated alongside individual datapoints, *n* = 4–6. Effects of 5 mg/kg once daily RXC004 versus vehicle on CD45^+^ score (**F**), dendritic cell (DC) score (**G**), or CXCL9 mRNA (**H**) in B16F10 tumors from C57BL/6 mice by Nanostring. Data are mean + SEM, *n* = 4–8. Statistical comparison; ordinary one-way ANOVA (**B**) or unpaired *t* test (**D**–**H**). *P* < 0.05 denoted by “*”, *P* < 0.01 denoted by “**” and *P* < 0.0001 as “****.” DC: dendritic cells, MDSC; myeloid-derived suppressor cells.

The B16F10/C57BL/6 melanoma syngeneic model is immune “cold” and nonresponsive to immune checkpoint therapy. We confirmed anti-PD-1 treatment had no effect on tumor volume, whereas significant tumor growth inhibition versus control animals was seen in response to RXC004 as either a monotherapy or in combination with anti-PD-1 (Fig. 5A and B, bodyweight data for the SNU1411 model and the B16F10 models are shown in Supplementary Fig. S5A and S5B). Similar findings were seen in combination with anti-CTLA-4 treatment in this model (Supplementary Fig. S6). RXC004 monotherapy also significantly increased survival in this B16F10/C57BL/6 model (Supplementary Fig. S5C). Moreover, RXC004 monotherapy efficacy was shown to be dose dependent and also achievable with a 5-day on/2-day off dosing schedule (Supplementary Fig. S6). When B16F10 cells were implanted into SCID-Beige mice, (an immune compromised strain), RXC004 treatment did not reduce tumor growth (Fig. 5C). This indicates that RXC004 exerts its antitumor effects in this model via suppressing immune evasion.

To confirm this proposed immune-related mechanism of action, flow cytometry studies were conducted in both B16F10 tumors and blood from tumor-bearing mice. RXC004 treatment significantly reduced tumor-associated MDSCs, with a concomitant increase in circulating MDSC in B16F10 tumor-bearing mice (Fig. 5D and E). Nanostring nCounter IO 360 analysis (23) indicated that RXC004 significantly increased the overall tumor-associated CD45^+^ immune cells, including dendritic cells, and increased tumor expression of the chemokine CXCL9 in this B16F10 model (Fig. 5F–H).

### RXC004 Effects on the Anti-PD-1 Immune Response in the CT26 “Hot” Model

**FIGURE 6:**
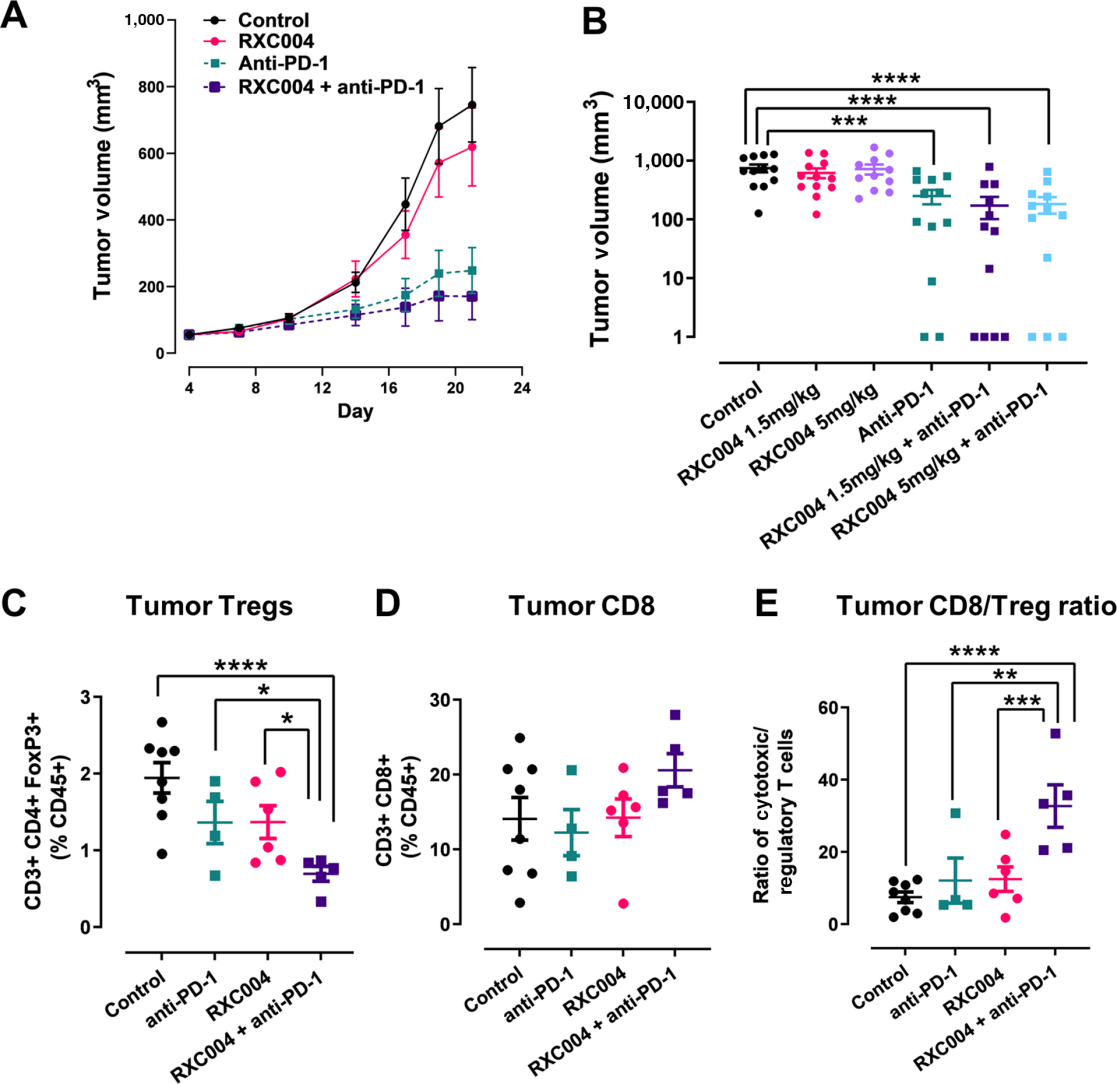
Effect of RXC004 as monotherapy or in combination with anti-PD-1 in the CT26/BALBc syngeneic model. **A,** Time course for vehicle, RXC004 (1.5 mg/kg once daily), anti-PD-1 antibody (10 mg/kg every 3 days), or RXC004 (1.5 mg/kg once daily) + anti-PD-1 (10 mg/kg every 3 days) combination treatment on tumor growth of CT26 xenografts in BALBc mice. Data are means ± SEM. **B,** Tumor volumes at day 21 in CT26/BALBc mice treated with vehicle, anti-PD-1 antibody (10 mg/kg, every 3 days), 1.5 mg/kg or 5 mg/kg once daily RXC004 alone or in combination with anti-PD-1 antibody. Mean ± SEM is indicated alongside individual datapoints, *n* ≥ 10. Effects of anti-PD-1, RXC004 (5 mg/kg once daily) or RXC004 + anti-PD-1 on tumor infiltration of regulatory T cells (Treg) (**C**), cytotoxic T cells (**D**), or cytotoxic T cell/Treg ratio (**E**) versus control. Mean ± SEM is indicated alongside individual datapoints, *n* ≥ 4. Statistical comparisons by ordinary one-way ANOVA. *P* < 0.05 denoted by “*”, *P* < 0.01 denoted by “**,” *P* < 0.001 denoted by “***,” and *P* < 0.0001 as “****.”

The CT26/BALBc colorectal syngeneic model is an immune “hot” model and, as expected, significant tumor growth inhibition was seen in response to anti-PD-1 treatment as a single agent (Fig. 6A and B). RXC004 monotherapy had limited effect on tumor growth in this model. Bodyweight data are shown in Supplementary Fig. S5D. However, analysis of CT26 tumor-infiltrating immune cells demonstrated a significant decrease in regulatory T cells (CD3^+^; CD4^+^; FOXP3^+^) when RXC004 was combined with anti-PD-1 treatment over control or either monotherapy. While a change in infiltrating CD8^+^ T cells was not observed, a significant increase in the CD8^+^/regulatory T cell ratio was seen for the RXC004 plus anti-PD-1 combination versus control or either monotherapy (Fig. 6C–E).

### RXC004 Treatment of HPAF-II Cells Abrogates Their Immune Suppression *In Vitro*

**FIGURE 7:**
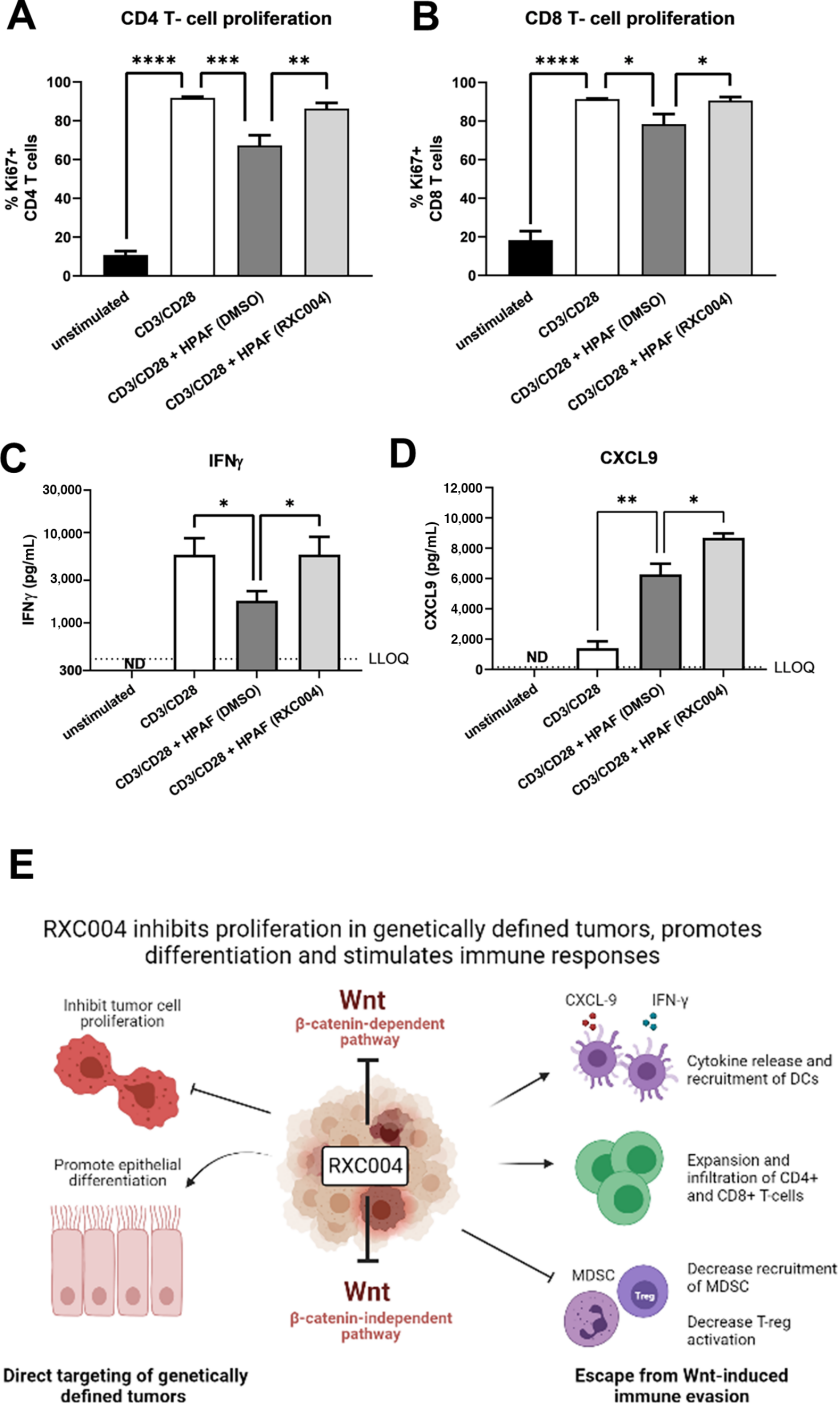
Effect of RXC004 on *in vitro* coculture of HPAF-II cells with human PBMCs. HPAF-II cells pretreated for 3 days with DMSO or RXC004 (100 nmol/L) were washed and reseeded at equivalent viable cell density before being cocultured with nonadherent human PBMCs stimulated by CD3/CD28 cross-linking. Proliferating populations of CD4 T cells (**A**) or CD8 T cells (**B**) were detected by Ki67 staining and flow cytometry. Data are means + SEM of four PBMC donors. Cytokine release of IFNγ (**C**) and CXCL9 (**D**) into the media of HPAF-II/PBMC cocultures was measured by Magpix multiplex analysis. Unstimulated and CD3/CD28-stimulated PBMC monocultures were used as negative and positive controls, respectively. Data are geomean + geometric SD for log transformed data (**C**) or mean + SEM (**D**) of four PBMC donors. Statistical comparisons were by RM one-way ANOVA. *P* < 0.05 denoted by “*”, *P* < 0.01 denoted by “**,” *P* < 0.001 denoted by “***,” and *P* < 0.0001 as “****.” ND: not detectable. LLOQ: Lower limit of quantification. **E,** Model of how RXC004 impacts genetically defined tumors by multiple mechanisms. Image created with BioRender.com, and adapted from Flanagan et al. PBMC: peripheral blood mononuclear cells.

*In vitro* coculture of control HPAF-II cancer cells with nonadherent human PBMCs significantly suppressed the CD3/CD28-induced proliferation of both CD8 and CD4 T cells (Fig. 7A and B), and also reduced levels of IFNγ and GMCSF (Fig. 7C; Supplementary Fig. S7A), cytokines important in generating an active antitumor immune response (24, 25). This ability of HPAF-II cells to inhibit T-cell proliferation and IFNγ/GMCSF release was fully reversed upon pretreatment of the cancer cells with RXC004 (Fig. 7A–C; Supplementary Fig. S7A). Furthermore, RXC004 pretreatment potentiated the HPAF-II–dependent release of the chemokine CXCL9 (Fig. 7D), while reducing the HPAF-II–dependent secretion of the angiogenesis promoting growth factor VEGF (Supplementary Fig. S7B). In nonadherent PBMC monoculture, recombinant Wnt3a or Wnt5a, when treated alone or in combination, had no significant effect on CD4 or CD8 T-cell proliferation or cytokine release (Supplementary Fig. S7C–S7F), suggesting that RXC004 effects in the coculture were unlikely to be solely via a direct reduction of Wnt levels. We therefore hypothesize that RXC004 treatment of HPAF-II cancer cells, and their resulting differentiation, has a secondary effect to reduce their immunosuppressive nature.

## Discussion

Dysregulation of canonical and noncanonical Wnt pathways plays a pivotal role in many gastrointestinal cancers (26). To date, attempts to target these pathways clinically have been limited to biological approaches, including the anti-Frizzled-7 antibody vantictumab and the decoy Frizzled receptor ipafricept, or small-molecule compounds with poorly defined mechanisms of action. Efficacy of the Frizzled-directed biologicals may be limited by redundancy in extracellular signaling, as these biologicals block only a subset of Wnt–Frizzled interactions. Furthermore, these clinical trials were performed in broad cancer patient populations, with no exploration of whether efficacy might be improved by preselection of patients with Wnt ligand–dependent disease. These biologicals have not performed well in the clinic to date, reporting dose-limiting toxicities, which may be due in part to their use in combination with standard-of-care chemotherapy regimens in many of these trials (7).

RXC004 is a potent small-molecule inhibitor of Porcupine that blocks Wnt palmitoylation, secretion, and downstream signaling, having a range of antitumor effects, as outlined in Fig. 7E. Direct tumor cell targeting was demonstrated by cell-cycle arrest, leading to reduced proliferation *in vitro* and inhibition of tumor growth in xenograft models. As predicted, antiproliferative effects were specific to models which retain a dependency on Wnt secretion, namely those driven by upstream Wnt pathway aberrations such as LoF mutations in *RNF43* or activating fusions in *RSPO2/3*. This is consistent with findings with other Porcupine inhibitors (27–29).

To explore the mechanism behind antiproliferative effects, we showed that RXC004 induced epithelial differentiation in genetically defined cancer cell lines with upstream Wnt pathway aberrations, as indicated by upregulation of MUC4 and MUC5AC mRNA and increased levels of mucin protein within cancer xenografts *in vivo.* This is consistent with findings with ETC-159, another Porcupine inhibitor, which extensively remodeled the transcriptome of colorectal cancer xenografts with *RSPO3* translocations, decreasing stem cell markers and promoting differentiation from adenocarcinoma to mucinous phenotype (30). Moreover, we demonstrated that, alongside increased differentiation, RXC004 reduced the glucose uptake in Wnt ligand–dependent cancer models, both *in vitro* and *in vivo* using ^18^FDG tracer. This suggests an opportunity to assess clinical activity of RXC004 in patients with genetically selected cancer by monitoring FDG-PET imaging in the clinic.

RXC004 effects were long lasting; RXC004-treated tumors reimplanted into naïve mice showed reduced tumor growth even in the absence of drug. This is consistent with findings with ETC-159, where tumor regrowth was suppressed for up to 6 weeks after withdrawal of treatment (30) and might be explained by the prodifferentiation effects of porcupine inhibition in these models. Long-lasting effects have also been observed in other situations were upstream Wnt signaling is blocked. Reengraftment of colorectal patient xenografts with *RSPO3* fusions from mice treated with anti-RSPO3 was significantly reduced, and regrowth inhibited even in the absence of continued anti-RSPO3 treatment (31). These findings of prolonged benefit after treatments have ceased could allow for more flexible dosing schedules for patients.

Reduced T-cell infiltration is associated with poor prognosis in colorectal and pancreatic cancers (32, 33) and canonical Wnt signaling is associated with downregulation of T-cell inflammation within the tumor microenvironment (34). Moreover, noncanonical Wnt5a has been shown to skew dendritic cells into a more tolerogenic state (35). We showed that, in coculture with human PBMCs, control *RNF43*-mutant HPAF-II pancreatic adenocarcinoma cells inhibit the stimulation of GMCSF and IFNγ production as well as CD4 and CD8 T-cell proliferation, effects that were reversed by preincubation of the HPAF-II cells with RXC004. RXC004 also potentiated release of CXCL9 from these cocultures, and reduced HPAF-II–dependent VEGF production. RXC004 also reduced MDSCs, while increasing overall immune infiltrate into the “cold tumor” B16F10 syngeneic model, which was confirmed to be nonresponsive to anti-PD-1 monotherapy, demonstrating a profound ability of RXC004 to reset aberrant immune responses, consistent with another recent study (36). In addition, CXCL9 mRNA expression was increased by RXC004 within the tumor microenvironment of this model. CXCL9 is a T-cell chemoattractant and its expression in tumors is linked to improved response to immune checkpoint inhibition in patients with cancer (37) and has also been associated with better prognosis in colorectal cancer (38).

The ability of RXC004 to inhibit tumor growth and enhance survival in the B16F10 model was dependent on a functional immune system, because neither effect was seen in B16F10 explants grown in immune-compromised SCID-Beige mice.

In the immune competent “hot tumor” CT26/BALBc syngeneic model, RXC004 synergized with anti-PD-1 treatment to decrease immunosuppressive regulatory T cells, and increase the ratio of cytotoxic CD8 T cells to regulatory T cells within the tumor. Higher CD8/regulatory T cell ratios in tumors have been shown to predict clinical benefit with anti-PD-1 therapy in patients with non—small cell lung cancer (39), hence our data support a rationale for combining RXC004 with anti-PD-1 therapies in the clinic.

Inducible ablation of β-catenin in the intestines of adult mice demonstrates the importance of canonical Wnt signaling in maintaining intestinal integrity (40). Given the pivotal role of Wnts in stem-cell renewal and homeostasis, the potential side effects of Porcupine inhibitors warrant investigation. High doses of Porcupine inhibitors have previously shown disruptive effects on villi architecture (41). We observed similar villi defects in mice at high doses of RXC004 but, importantly, profound effects of RXC004 on tumor growth and immune infiltrate were seen at lower doses that had no impact on intestinal homeostasis, confirming previous observations that a therapeutic window exists for this class of inhibitors (42). This window may be due to several factors; the high expression of drug exporters in stromal cells of the lower crypt, the ability of different populations of crypt stem cells to compensate for each other, and/or the existence of a Wnt gradient potentially protecting basal crypt stem cells from the effects of Porcupine inhibitors (43–45). Moreover, the ability of the intestinal epithelium to recover from insult has been well established; for example, restoration of functional APC in animal colorectal cancer models led to resumption of normal crypt-villus homeostasis, with aberrantly proliferating cells reacquiring self-renewal and multilineage differentiation capability (46).

Several Porcupine inhibitors, including RXC004, are currently being investigated in the clinic. These include WNT974 (previously LGK974), CGX1321, ETC-159, and XNW7201. Encouragingly, there has been a lack of widespread intestinal adverse events published to date. The most consistent side effects include loss of bone density and associated fractures, and dysgeusia (47), the latter likely due the role of Wnts in the renewal of taste buds (48). Bone effects of Porcupine inhibitors can be mitigated in animals by prophylactic treatment with RANKL inhibitors or bisphosphonates (49), an approach that has also proved effective in the clinic (50). An advantage of small-molecule inhibitors such as RXC004 compared with biologicals such as ipafricept and vantictumab is the ability to explore subtly different dosing strategies. It is interesting to note the differences in dosing regimens currently being explored with Porcupine inhibitors, ranging from priming dose only for WNT974 (when in combination with an anti-PD-1 therapy), to once daily (RXC004), or once every 2 days (ETC-159; refs. 51–53). The impact of these different dosing schedules on efficacy and safety is yet to be determined.

Our observations that Porcupine inhibitors are effective in a subset of genetically defined cancer models suggest that prospective selection of patients with Wnt ligand–dependent cancers will be required. Examples include patients with colorectal or pancreatic cancer with *RSPO2/3* fusions and LoF *RNF43* mutations. Because of these genetically selected patient tumors likely being dependent on Wnt ligand for survival and growth, it follows that high Wnt ligand levels in the tumor microenvironment could also be responsible for increased immunosuppression. This may increase susceptibility of such patient tumors to the positive immune effects of Porcupine inhibitors such as RXC004. The synergistic effect between RXC004 and anti-PD-1 on immune infiltrate in the “immune hot” CT26 model and the reversal of immune suppression in the “immune-cold” B16F10 model supports investigating the clinical activity of Porcupine inhibitors in combination with anti-PD-1 therapy, a strategy currently being explored (47). Investigation of Porcupine inhibitors in other gastrointestinal cancers may also be of interest. For example, in biliary cancer, Wnt ligands are reported to be highly expressed and associated with poor prognosis. In addition there are reports of hypermethylation-induced silencing of extracellular Wnt pathway antagonists (54, 55).

In summary, the Porcupine inhibitor RXC004 shows potent, antiproliferative, prodifferentiation, and immune-stimulating effects in colorectal, pancreatic, and syngeneic cancer models at doses that have no effect on normal intestinal integrity. RXC004 is currently undergoing clinical trials in patients with cancer as a monotherapy and in combination with anti-PD-1 therapy.

## Supplementary Material

Supplementary Figures 1-7, Table 1 Supplementary Figure 1: Effect of RXC004 on gene expression, caspase activity and cell growth in colorectal and pancreatic cancer cell lines in vitro. Supplementary Figure 2: RXC004 effects on gene expression in in vivo xenograft tumor models. Supplementary Figure 3: Pharmacokinetic/pharmacodynamic (PK/PD) analysis of RXC004 in SNU-1411/NOD-SCID mice. Supplementary Figure 4: Dose-dependent effects of RXC004 on tumor markers, intestinal Ki67 and bodyweight. Supplementary Figure 5: Effect of RXC004 with or without anti-PD-1 on body weight and survival in in vivo models. Supplementary Figure 6: Dose-dependent effects of RXC004 alone or in combination with anti-CTLA-4 in B16F10/C57BL/6 syngeneic model. Supplementary Figure 7: In vitro PBMC co-culture additional cytokines and monocultures. Supplementary Table 1: In vivo exposure data for RXC004.
