# Supplementary material for: The Wnt Pathway Inhibitor RXC004 Blocks Tumor Growth and Reverses Immune Evasion in Wnt Ligand–dependent Cancer Models
Source: Cancer Res Commun. 2022 Sep 2;2(9):914–28. doi: 10.1158/2767-9764.CRC-21-0095 (PMC10010340; doi:10.1158/2767-9764.CRC-21-0095)
Supplement: Supplementary Figures 1-7, Table 1 — Supplementary Figure 1: Effect of RXC004 on gene expression, caspase activity and cell growth in colorectal and pancreatic cancer cell lines in vitro. Supplementary Figure 2: RXC004 effects on gene expression in in vivo xenograft tumor models. Supplementary Figure 3: Pharmacokinetic/pharmacodynamic (PK/PD) analysis of RXC004 in SNU-1411/NOD-SCID mice. Supplementary Figure 4: Dose-dependent effects of RXC004 on tumor markers, intestinal Ki67 and bodyweight. Supplementary Figure 5: Effect of RXC004 with or without anti-PD-1 on body weight and survival in in vivo models. Supplementary Figure 6: Dose-dependent effects of RXC004 alone or in combination with anti-CTLA-4 in B16F10/C57BL/6 syngeneic model. Supplementary Figure 7: In vitro PBMC co-culture additional cytokines and monocultures. Supplementary Table 1: In vivo exposure data for RXC004. [file crc-21-0095-s01.docx]

**Supplemental Figure 1: Effect of RXC004 on gene expression, caspase activity and cell growth in colorectal and pancreatic cancer cell lines *in vitro*.** A-D: RXC004 (10nM) effects on mRNA expression on the Wnt-ligand dependent JVE-109 (A), AsPC1 (B) and CAPAN-2 (C) cell lines, and the Wnt-ligand independent HCT-116 cell line (D). Data presented are representative experiments, showing fold differential mRNA expression versus DMSO controls. E: Concentration-dependent effect of RXC004 on c-Myc mRNA expression in colorectal and pancreatic cell lines. Data is mean and SEM of IC_50_ values for ≥3 experiments. WiDR and HCT116 IC_50_ values were >10µM. F, G: RXC004 (100nM) treatment for 7 days has no effect on relative Caspase 3/7 activity in SNU-1411 or HPAF-II cells. Data is Mean +SEM from *n*=3. H, I: *In vitro* IncuCyte growth curves over time in SNU-1411 or HPAF-II cells in presence or absence of 100nM RXC004. Data is single, representative experiment.


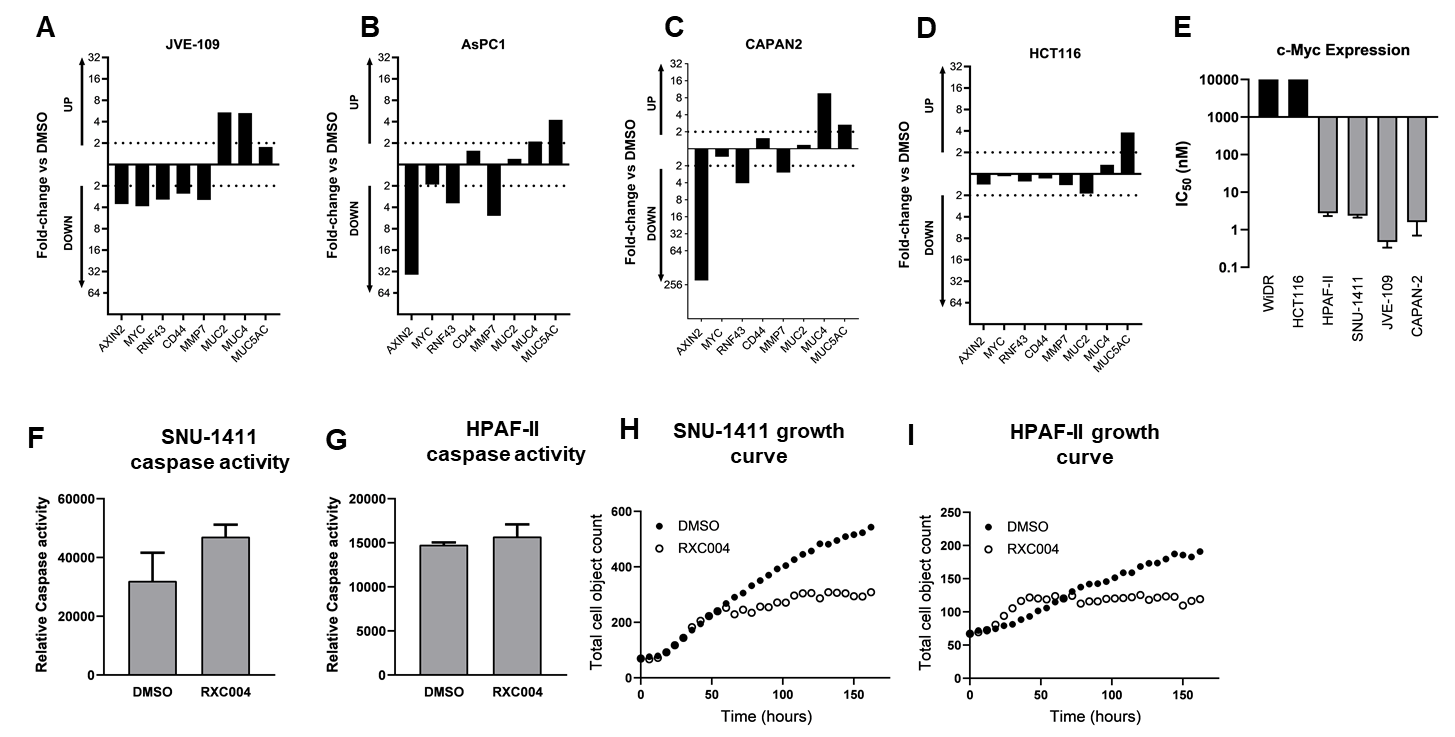


**Supplemental Figure 2: RXC004 effects on gene expression in *in vivo* xenograft tumor models.** A, G, M, P: RXC004 was dosed orally at 1.5mg/kg BID in *in vivo* xenograft models, effects on relative tumor volume (HPAF-II (A), SNU-1411 (M) and HCT116 (P)), or tumor weight (AsPC1 (G)) are shown. Mean ± SEM is indicated alongside individual datapoints B-F: Effect of vehicle or RXC004 (1.5mg/kg BID) on c-Myc, Axin-2, RNF43, MUC5AC and MUC4 mRNA expression in HPAF-II xenografts. H-L: Effect of vehicle or RXC004 (1.5mg/kg BID) on c-Myc, Axin-2, RNF43, MUC5AC and MUC4 mRNA expression in AsPC1 xenografts. N-R: Effect of vehicle or RXC004 (1.5mg/kg BID) on c-Myc, Axin-2, RNF43, MUC5AC and MUC4 mRNA expression in SNU-1411 xenografts. Data are Mean + SEM, *n=8* tumors (from 4 animals with bilateral tumors). Statistical comparisons by Mann-Whitney U for tumour weight and volume analyses, and by unpaired T-test for gene expression. *P* <0.05 denoted by “*”, *P* <0.01 denoted by “**”, *P* <0.001 denoted by “***”, and *P* <0.0001 denoted by “****”.


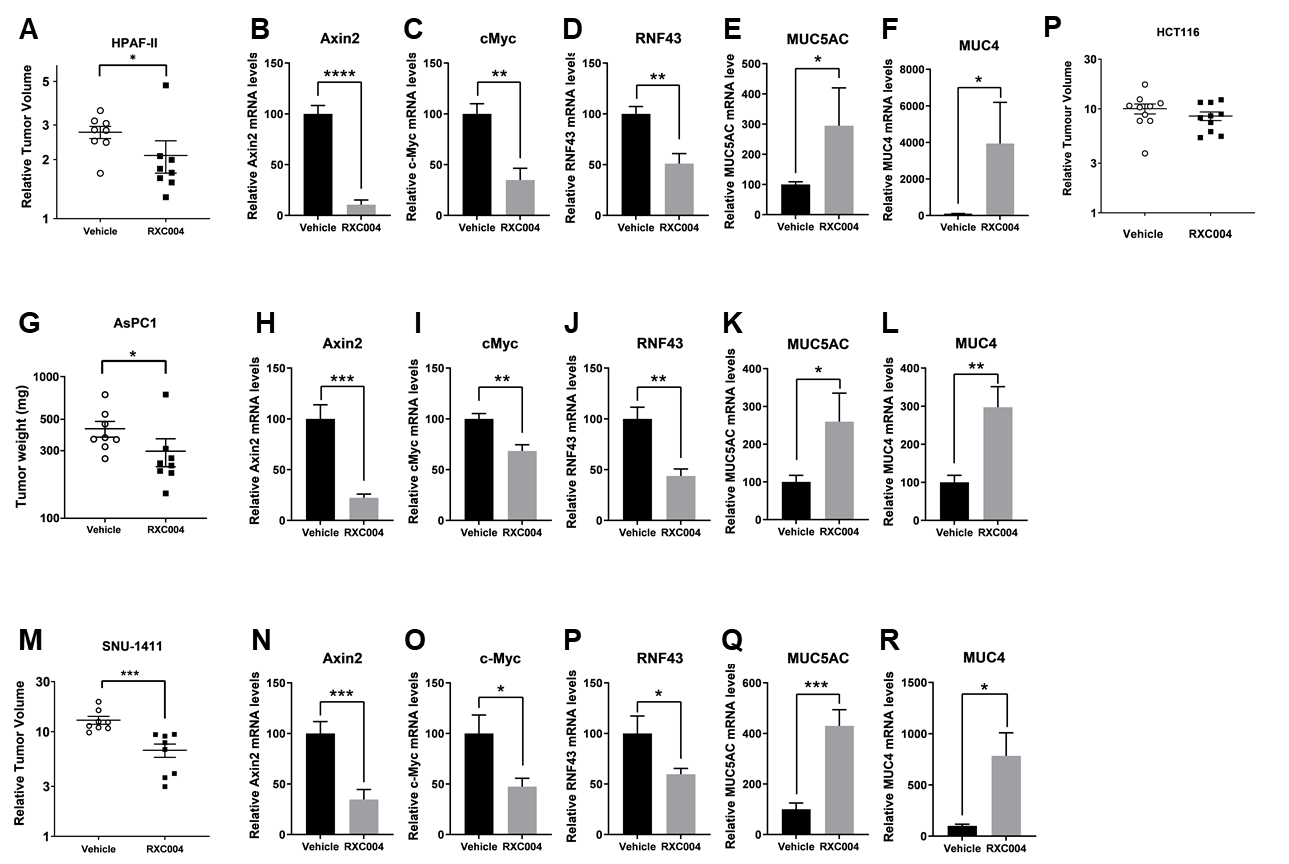


**Supplemental Figure 3. Pharmacokinetic/pharmacodynamic (PK/PD) analysis of RXC004 in SNU-1411/NOD-SCID mice.** RXC004 was dosed at 1.5mg/kg QD for 7 days, drug levels in tumor and plasma were analysed post final dose (A), data are mean ± SEM, *n*=4 per timepoint. Corresponding tumor mRNA expression of Wnt responsive genes at indicated time points post final RXC004 dose (B-F), mean ± SEM is indicated alongside individual datapoints. RXC004 was dosed at 5mg/kg QD for 7 days, drug levels in tumor and plasma were analysed post final dose (G), data are mean ± SEM, *n*=4 per timepoint. Corresponding tumor mRNA expression of Wnt responsive genes at indicated time points post final RXC004 dose (H-L), mean ± SEM is indicated alongside individual datapoints. Statistical comparisons versus vehicle group; ordinary one-way ANOVA. *P* <0.05 denoted by “*”, *P* <0.01 denoted by “**”, *P* <0.001 denoted by “***” and *P* <0.0001 denoted by “****”.


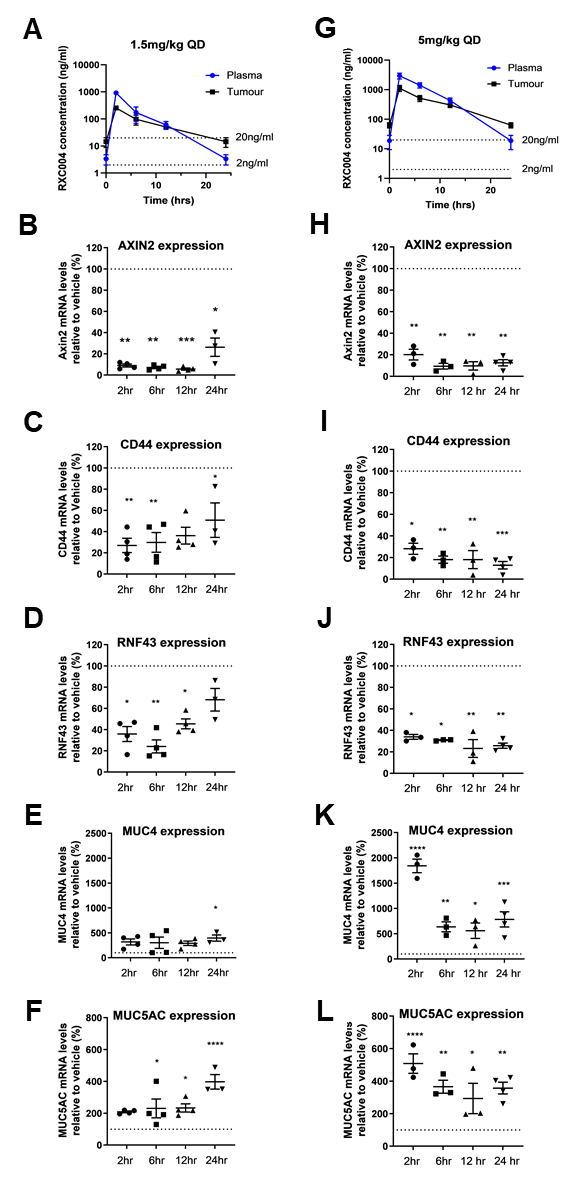


**Supplemental Figure 4: Dose-dependent effects of RXC004 on tumor markers, intestinal Ki67 and bodyweight**. A: Quantification of Mucin (as stained by AB_PAS) in SNU-1411 tumor tissue following 7 days of the indicated treatment. Data is geometric mean + SD, *n*=3; statistical comparisons of log transformed data by ordinary one-way ANOVA. B: Percentage of tumor (SNU-1411 model) classified as differentiated after treatment for 7 days with 1.5 mg/kg or 5 mg/kg QD RXC004, data is mean + SEM, *n*=3. Differentiated tumor could not be classified in vehicle treated animals. C: Ki67 quantification per mm^2^ of total or differentiated tumor in SNU-1411 model following 7 days of the indicated treatment. Data is mean + SEM, *n*=3; statistical comparisons by ordinary one-way ANOVA. *P* <0.05 denoted by “*”, *P* <0.01 denoted by “**”. D-F: SCID-Beige mice were treated for 28 consecutive days with vehicle (D), RXC004 at 1.5mg/kg BID (E), or RXC004 at 5mg/kg BID (F). Ki67 staining by immunohistochemistry (brown stain). 20x magnification. G: Body weight (means ± SEM) over time in SCID-Beige mice treated with the indicated doses of RXC004.


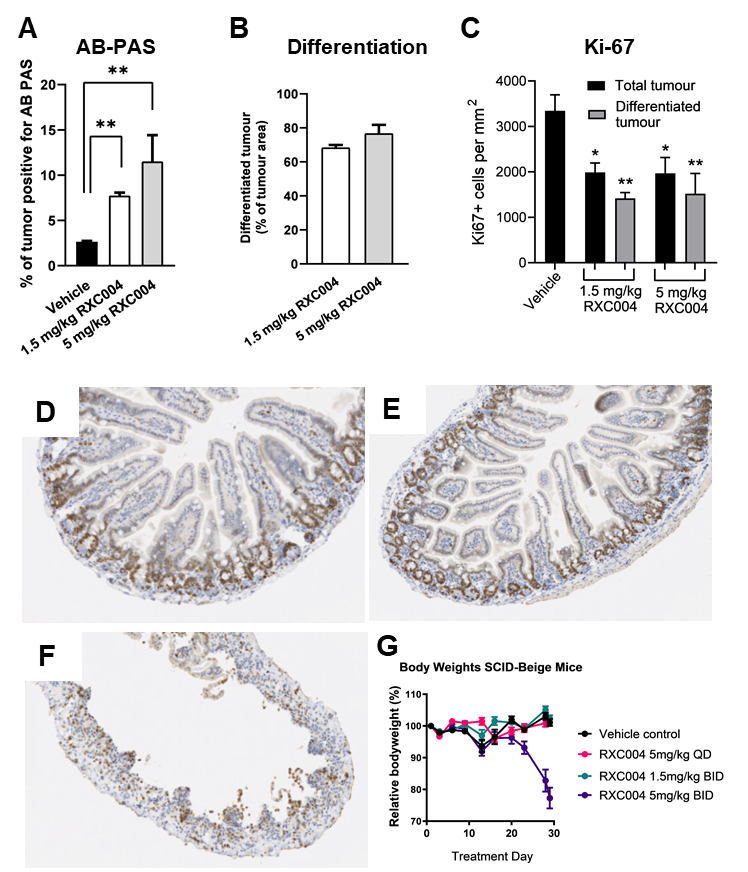


**Supplemental Figure 5: Effect of RXC004 with or without anti-PD-1 on body weight and survival in *in vivo* models**

A: Effect of various RXC004 dosing regimens on body weight change in SNU-1411-implanted NOD-SCID mice (same study as shown in Figure 3A). QD, once daily; BID, twice daily; 5/2, 5 days dosing interspersed with 2 days without dosing. Data is means ± SEM. B: Effect of vehicle, RXC004 (1.5mg/kg QD), anti-PD-1 antibody (100µg per dose, BIW), or RXC004 (1.5mg/kg QD) + anti-PD-1 combination treatment on body weight of B16F10/C57BL/6 mice (same study as shown in Figure 5A). *n* =10 per group, data is means ± SEM. C: Kaplan-Meier survival curve in C57BL6/B16F10 mice in response to vehicle or 5 mg/kg QD RXC004. Data is from same study as Figure 5A, 5B and panel C. Significant survival benefit of RXC004 treatment was determined (P <0.0001; Log-rank test) with a hazard ratio of 0.27. D: Effect of vehicle, RXC004 (1.5mg/kg QD), anti-PD-1 antibody (10mg/kg, Q3D), or RXC004 (1.5mg/kg QD) + anti-PD-1 combination treatment on body weight in CT26/BALBc mice (same study as shown in Figure 6A). Data is means ± SEM.

**
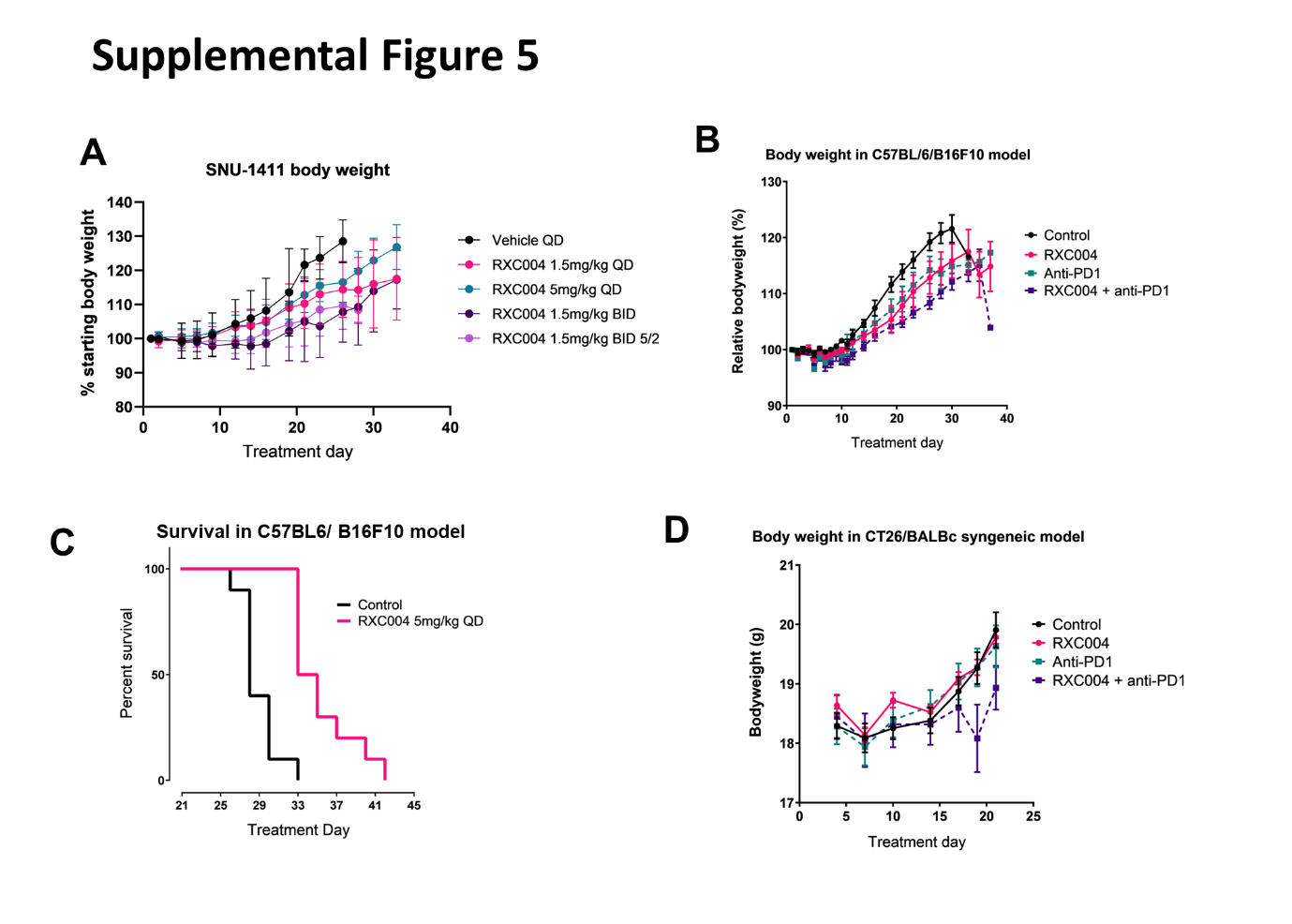
**

**Supplemental Figure 6: Dose-dependent effects of RXC004 alone or in combination with anti-CTLA-4 in B16F10/C57BL/6 syngeneic model.** A: Time course of RXC004 effects on tumor volume in B16F10/C57BL/6 xenografts with various indicated dosing regimens of RXC004. QD: once daily, QoD: once every other day, 5 on/2 off: five days dosing with 2 days non-dosing (indicated by grey bars). Data is mean ± SEM. B: Individual animal day 27 tumor volumes from panel A in B16F10/C57BL/6 xenografts following different RXC004 dosing regimens, mean ± SEM is indicated alongside individual datapoints. C: Time course/dose-response of RXC004 alone on tumor volume in B16F10/C57BL/6 xenografts (all RXC004 doses QD) or in combination with anti-CTLA-4. Data is mean ± SEM. D: Individual day 27 tumor volumes from panel C following treatment with RXC004 alone (1.5 or 5mg/kg QD) or in combination with anti-CTLA-4. Mean ± SEM is indicated alongside individual datapoints, *n* =14-15 for controls, *n* = 10-12 for all other groups. Statistical comparisons by ordinary one-way ANOVA versus vehicle (B) or as indicated (D). *P* <0.05 denoted by “*”, *P* <0.01 denoted by “**”, *P* <0.001 denoted by “***” and *P* <0.0001 as “****”.


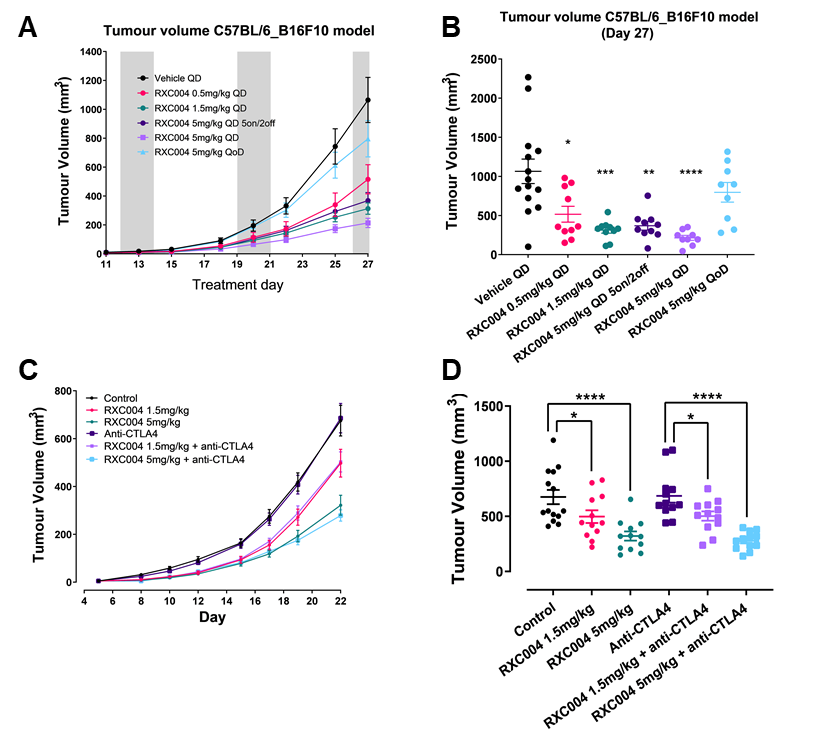


**Supplemental Figure 7: *In vitro* PBMC co-culture additional cytokines and monocultures**

Effect of RXC004 (100nM) pre-treatment of HPAF-II cells on GM-CSF (A) and VEGF (B) release by HPAF-II/PBMC co-cultures stimulated by CD3/CD28 cross-linking. C, D: Wnt3a and/or Wnt5a (100 ng/ml) do not directly impact CD4 or CD8 T-cell proliferation in PBMC monocultures. E, F, G: Wnt3a and/or Wnt5a do not directly affect IFNγ or GM-CSF secretion from PMBC monocultures. In all cases, unstimulated and CD3/CD28-stimulated PBMC monocultures were used as negative and positive controls respectively. Data is means + SEM (C, D), or geometric mean + geometric SD for log transformed data (A, B, E, F). n = 4 PBMC donors, except for panel F (n=3); statistical comparisons were by RM one way ANOVA (A, C-F), paired T-test (B). *P* <0.05 denoted by “*”, *P* <0.01 denoted by “**”, *P* <0.001 denoted by “***”. ND: not detectable. LLOQ: Lower limit of quantification.

**
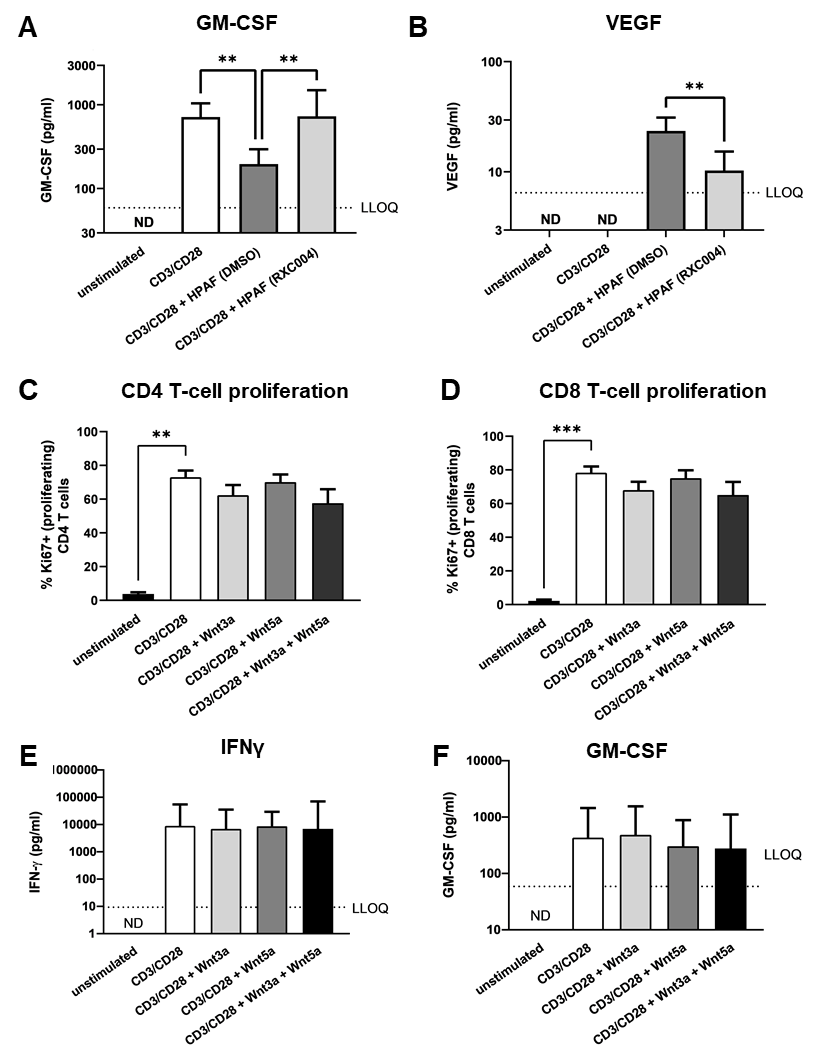
**

**Supplemental Table 1: *In vivo* exposure data for RXC004**

| species | Dose (i.v./p.o., mg/kg) | C_max_ (p.o., µM) | C_24h_ (p.o., µM) | AUC*_inf_* (p.o., µM.h) | Cl (mL/min/kg) | Vss (L/kg) | F (p.o., %) | T_½_ (hr) |
| --- | --- | --- | --- | --- | --- | --- | --- | --- |
| mouse*^a^* | 2/5 | 7.6 | 0.002 | 33.9 | 2.9 | 0.40 | 48 | 1.8 |
| rat*^b^* | 2/5 | 3.6 | 0.009 | 10.5 | 5.8 | 0.64 | 31 | 2.5 |
| dog*^c^* | 2/5 | 10.4 | 0.012 | 8.6 | 8.9 | 0.39 | 137 | 0.8 |

*^a^*Formulation (i.v.), 5% DMSO: 95%HPBC (25% w/v); formulation (p.o.), 0.5% CMC and 0.1% Tween-80

*^b^*Formulation (i.v.), 5% DMSO: 95%HPBC (25% w/v); formulation (p.o.), 0.5% HPMC

*^c^*Formulation (i.v.), 5% DMSO: 50%PEG 400 (v/v); formulation (p.o.), 5% DMSO: 95%HPBC (25% w/v)
